# Real-world Chinese herbal medicine for Parkinson's disease: a hospital-based retrospective analysis of electronic medical records

**DOI:** 10.3389/fnagi.2024.1362948

**Published:** 2024-05-02

**Authors:** Shaohua Lyu, Claire Shuiqing Zhang, Zhenhui Mao, Xinfeng Guo, Zhe Li, Xiaodong Luo, Jingbo Sun, Qiaozhen Su

**Affiliations:** ^1^The Second Affiliated Hospital of Guangzhou University of Chinese Medicine, Guangdong Provincial Hospital of Chinese Medicine, Guangdong Provincial Academy of Chinese Medical Sciences, Guangzhou, China; ^2^School of Health and Biomedical Sciences, STEM College, RMIT University, Bundoora, VIC, Australia

**Keywords:** Parkinson's disease, electronic medical records, real-world study, Chinese herbal medicine, Chinese medicine, *Bu zhong yi qi tang*

## Abstract

**Background:**

Parkinson's disease (PD) is a progressive neurodegenerative condition. Chinese medicine therapies have demonstrated effectiveness for PD in controlled settings. However, the utilization of Chinese medicine therapies for PD in real-world clinical practice and the characteristics of patients seeking these therapies have not been thoroughly summarized.

**Method:**

The study retrospectively analyzed initial patient encounters (PEs) with a first-listed diagnosis of PD, based on electronic medical records from Guangdong Provincial Hospital of Chinese Medicine between July 2018 and July 2023.

**Results:**

A total of 3,206 PEs, each corresponding to an individual patient, were eligible for analyses. Approximately 60% of patients made initial visits to the Chinese medicine hospital after receiving a PD diagnosis, around 4.59 years after the onset of motor symptoms. Over 75% of the patients visited the Internal Medicine Outpatient Clinic at their initial visits, while a mere 13.85% visited PD Chronic Care Clinic. Rest tremor (61.98%) and bradykinesia (52.34%) are the most commonly reported motor symptoms, followed by rigidity (40.70%). The most commonly recorded non-motor symptoms included constipation (31.88%) and sleep disturbance (25.27%). Integration of Chinese medicine and conventional medicine therapies was the most common treatment method (39.15%), followed by single use of Chinese herbal medicine (27.14%). The most frequently prescribed herbs for PD included *Glycyrrhiza uralensis* Fisch. (*gan cao*), *Astragalus mongholicus* Bunge (*huang qi*), *Atractylodes macrocephala* Koidz. (*bai zhu*), *Angelica sinensis* (Oliv.) Diels (*dang gui*), *Rehmannia glutinosa* (Gaertn.) DC. (*di huang*), *Paeonia lactiflora* Pall. (*bai shao*), *Bupleurum chinense* DC. (*chai hu*), *Citrus aurantium* L. (*zhi qiao*/*zhi shi/chen pi*), *Panax ginseng* C. A. Mey. (*ren shen*), and *Poria cocos* (Schw.) Wolf (*fu ling*). These herbs contribute to formulation of *Bu zhong yi qi tang* (BZYQT).

**Conclusion:**

Patients typically initiated Chinese medical care after the establishment of PD diagnosis, ~4.59 years post-onset of motor symptoms. The prevalent utilization of CHM decoctions and patented Chinese herbal medicine products, underscores its potential in addressing both motor and non-motor symptoms. Despite available evidence, rigorous clinical trials are needed to validate and optimize the integration of CHM, particularly BZYQT, into therapeutic strategies for PD.

## 1 Introduction

Parkinson's disease (PD) is a progressive, neurodegenerative disorder characterized by core motor symptoms, collectively known as Parkinsonism. These symptoms typically include bradykinesia, marked by slow movement, and are often accompanied by rest tremor or rigidity (Postuma et al., 2015). Alongside these motor symptoms, PD presents a spectrum of non-motor symptoms such as rapid eye movement sleep behavior disorder (RBD), constipation and depression (Mehndiratta et al., 2011). Non-motor symptoms can manifest at any stage of PD, sometimes serving as prodromal signs preceding motor symptoms (National Institute of Neurological Disorders and Stroke, 2004; Lee and Koh, 2015). Both motor and non-motor symptoms significantly impact the quality of life for individuals with PD (Santos García et al., 2019), with non-motor symptoms sometimes becoming the primary complaints prompting medical visits (Frucht, 2004; O'Sullivan et al., 2008). According to a systematic review of the Global Burden of Disease (GBD) 2016, PD affected ~6.1 million people worldwide, resulting in 3.2 million disability adjusted life years (DALYs) (GBD 2016 Parkinson's Disease Collaborators, 2018). PD typically emerges in individuals aged over 50, and its prevalence increased with age (GBD 2016 Parkinson's Disease Collaborators, 2018). By 2019, China had become one of the top five countries with the highest prevalence of PD cases and associated DALYs (Zhong and Zhu, 2022), with projections indicating a continuous rise in both prevalence and DALYs (Chen et al., 2022).

Various antiparkinsonian medications have been developed for the management of PD, including levodopa (either alone or with a dopa decarboxylase inhibitor), dopamine agonists, monoamine oxidase-B (MAO-B) inhibitors, catechol-O-methyl transferase (COMT) inhibitors, anticholinergics, and N-methyl-D-aspartate (NMDA) receptor antagonists (National Institute for Health and Care Excellence, 2017; Grimes et al., 2019; Parkinson's Disease and Movement Disorders Group from Neurology Branch of Chinese Medical Association and Parkinson's Disease and Movement Disorders Group from Neurology Branch of Chinese Medical Doctor Association, 2020; Pringsheim et al., 2021; Waller et al., 2021). However, these pharmacotherapies often come with inevitable side effects. For instance, dopamine agonists, considered a first-line treatment, may exacerbate certain non-motor symptoms such as impulse control disorders, excessive sleepiness, and psychotic symptoms (National Institute of Neurological Disorders and Stroke, 2004; National Institute for Health and Care Excellence, 2017). Another first-line medication, Levodopa, often leads to motor complications like dyskinesia, motor fluctuations, and “wearing off” phenomena during the middle-to-late stages of PD (National Institute of Neurological Disorders and Stroke, 2004; National Institute for Health and Care Excellence, 2017), imposing significant burdens on patients (Santos-García et al., 2020).

Despite the availability of adjunctive pharmacotherapies and surgical interventions for motor complications and non-motor symptoms, adjunctive pharmacotherapies have limitations in clinical effectiveness with additional side effects (Waller et al., 2021). Moreover, surgery is often contraindicated in elderly patients with advanced PD (Dewey, 2004). There is an unmet need for effective and safe treatments to assist conventional antiparkinsonian strategies, which may enhance clinical effectiveness in controlling motor and non-motor symptoms throughout PD course, minimize the risks of medication-induced motor complications in early stages of PD, and improve management of motor complications in advanced stages (Dewey, 2004; LeWitt and Chaudhuri, 2020; Rukavina et al., 2021).

In light of the challenges outlined above, an increasing number of PD patients tend to seek complementary and alternative therapies, such as herbal medicine, acupuncture, and other modalities, to enhance and complement their anti-Parkinson's management (Rajendran et al., 2001; Ferry et al., 2002; Tan et al., 2006; Kim et al., 2009; Lökk and Nilsson, 2010; Pecci et al., 2010). Particularly noteworthy is the popularity of traditional herbal medicine, especially Chinese herbal medicine (CHM), among Asian PD patients (Tan et al., 2006; Kim et al., 2009; Lin et al., 2021). CHM, deeply rooted in a history spanning thousands of years in China, places emphasis on individualized syndrome differentiation (Li et al., 2011). Although clinical guidelines recommend Chinese medicine therapies for PD, encompassing the treatment of both motor and non-motor symptoms, as well as motor complications (Cho et al., 2018; Liu et al., 2020; Li W. et al., 2021; Luo et al., 2021; Yang et al., 2021; Zhao and Liu, 2021; Yun and Liu, 2022), it is acknowledged that certain guideline recommendations lack robust evidence from high-quality research (Liu et al., 2020; Zhao and Liu, 2021). While some recent guidelines derive their clinical recommendations from evidence obtained through randomized controlled trials (RCT) and RCT-based systematic reviews, such evidence often faces constraints in terms of generalizability and clinical applicability (Green and Glasgow, 2006; Sanson-Fisher et al., 2007). In controlled settings, PD patients are typically prescribed standardized formulas including *Ping chan* granule, *Cong rong shu jing* granules, and *Hua tan jie yu* granules (Chen et al., 2020; Liu et al., 2020a; Gu et al., 2023). However, these formulas were usually tailored to specific patients and may not effectively address the diverse symptoms of PD patients in real-world situations. Real-world clinical practice experiences are crucial for informing evidence-based approaches to treating PD with Chinese medicine (Black, 1996; Dreyer, 2022). Furthermore, the clinical characteristics of PD patients who seek Chinese medicine, and when they start to seek Chinese medicine therapies for PD remained unclear. Given the complexity of PD symptoms and complications, it is important to understand patients' primary concerns, the symptoms that most bother them, and their treatment preferences. As a fundamental component of evidence-based practice, patients' preferences and values deserves in-depth exploration to optimize Chinese medicine treatments for individuals with PD.

To address these gaps, we conducted a retrospective analysis of electronic medical records (EMRs) from a tertiary Chinese medicine hospital. The aim was to explore and summarize real-world clinicians' experiences in prescribing Chinese medicine to PD patients, and identify the characteristics of PD patients receiving initial CHM treatments. The insights gained from this analysis will contribute valuable information to support evidence-based clinical practice of Chinese medicine for PD.

## 2 Methods

The study collected and analyzed data of the existing EMRs from outpatient departments at Guangdong Provincial Hospital of Chinese Medicine (GPHCM), a tertiary hospital providing integrated Chinese and conventional medicine for PD patients in China (Guangdong Provincial Hospital of Chinese Medicine, 2021). The study proposal was reviewed and approved by the Human Research Ethics Committee (HREC) of GPHCM (ZE2023-392-01) with waived informed consent.

### 2.1 Data search and screening

Outpatient EMRs with a first-listed diagnosis of PD, whether confirmed or suspected, were identified in the electronic EMR system of GPHCM between July 2018 and June 2023. Only patient encounters (PEs) for the initial medical visits for Parkinsonism were retrieved out of these EMRs, and exported to an Excel sheet, with assistance provided by the Information Technology Department of GPHCM.

Eligibility screening was carried out by Shaohua Lyu, a clinician specializing in PD and neurological conditions. Follow-up PEs with initial encounters outside the research timeframe and initial PEs lacking detailed descriptions of medical history (including symptoms) were excluded. Any uncertainty was resolved through consultation with a senior PD specialist (X Luo or Q Su).

### 2.2 Status of diagnosis

As medical diagnosis may or may not have been definitively established during the initial visit (CAER Inc, 2023), the status of the first-listed PD diagnosis at the initial visit was further classified into three categories: (1) A “confirmed diagnosis,” if the patient had received a formal PD diagnosis before visiting the studied hospital; (2) A “suspected diagnosis,” if the patient's symptoms and complaints were indicative of parkinsonism, but a confirmed PD diagnosis had not been established at the initial visits; (3) An “unclear status of diagnosis,” when there was insufficient information to determine whether a PD diagnosis has been established from the initial PEs (Shah et al., 2019).

### 2.3 Data extraction

General information such as age, disease duration (time from the onset of motor symptoms), onset age, and gender, visited departments, typical motor symptoms, common motor complications, and non-motor symptoms along with details of prescriptions including herb ingredients of prescribed CHM decoctions, names and herb ingredients of patented Chinese herbal medicine products (PCHMPs), acupuncture, and names of antiparkinsonian medications, were extracted by Shaohua Lyu and double-checked by Zhenhui Mao.

Extracted motor symptoms included bradykinesia, rest tremor, rigidity and postural instability (Postuma et al., 2015). Motor complications comprised dyskinesia and motor fluctuations, which encompassed the “on-off” phenomenon and/or “wearing off” (Freitas et al., 2017). These complications may either signify the progressive degeneration of nigrostriatal dopaminergic neurons in nature or result from levodopa-induced side effects (Kim et al., 2020). Non-motor symptoms extracted during this study encompassed constipation, musculoskeletal pain, fatigue, orthostatic hypotension, restless legs, sweating, swallowing dysfunction, salivation, cognitive impairment, urinary problems, hallucinations and delusions, anxiety and/or depression, excessive daytime sleepiness, and sleep disturbance (including RBD) (Chaudhuri et al., 2007; Carroll et al., 2021).

### 2.4 Data standardization

Diverse descriptions of the same PD symptom in the EMRs text were standardized using common medical terms. For instance, rigidity in arms, legs or neck were standardized as rigidity, irrespective of the specific locations mentioned in the text. Herbs being processed in different ways were also standardized. For example, *zhi huang qi* (fried *huang qi*) was simplified as *huang qi* as no distinction was observed in their nature. A similar approach was taken with *gan cao* and *zhi gan cao*, as well as *zhi qiao* and *chao zhi qiao*. It should be noted that *zhi qiao, zhi shi* and *chen pi* are all fruit peels collected at various stages from the same plant (*Citrus aurantium* L.). However, the former two share a similar function, while *chen pi* has a distinguishing role in Chinese medicine theory and was separated from the other two herbs during frequency analysis. Scientific names commonly used Latin names and traditional Chinese names of the herbs involved in this study are introduced in Table 1.

**Table 1:** Frequency of commonly used herbs for Parkinson's disease.

Scientific names^*^	**Latin names**	**Chinese names in *Pinyin***	**Frequency (%) (Total *n =* 1,764)**
1. *Glycyrrhiza uralensis* Fisch. 2. *Glycyrrhiza inflata* Batalin 3. *Glycyrrhiza glabra* L.	*Glycyrrhizae Radix et* Rhizoma	*Gan cao^#^*	1,252 (70.98)
*Astragalus mongholicus* Bunge	*Astragali* Radix	*Huang qi^#^*	953 (54.02)
*Atractylodes macrocephala* Koidz.	*Atractylodis Macrocephalae* Rhizoma	*Bai zhu^#^*	948 (53.74)
*Angelica sinensis* (Oliv.) Diels	*Angelicae* Sinensis Radix	*Dang gui^#^*	844 (47.85)
*Rehmannia glutinosa* (Gaertn.) DC.	*Rehmanniae* Radix	*Di huang^#^*	753 (42.69)
*Paeonia lactiflora* Pall.	*Paeoniae* Radix Alba	*Bai shao*	614 (34.81)
1. *Bupleurum chinense* DC. 2. *Bupleurum scorzonerifolium* Willd.	*Bupleuri* Radix	*Chai hu*	564 (31.97)
*Citrus aurantium* L.	*Aurantii* Fructus	*Zhi qiao*/*Zhi shi*	557 (31.58)
*Panax ginseng* C. A. Mey.	*Ginseng* Radix et Rhizoma	*Ren shen^#^*	502 (28.46)
*Citrus aurantium* L.	*Citri Reticulatae* Pericarpium	*Chen pi^#^*	501 (28.40)
*Poria cocos* (Schw.) Wolf	Poria	*Fu ling^#^*	480 (27.21)
1. *Codonopsis pilosula* Nannf. 2. *Codonopsis pilosula var*. pilosula 3. *Campanumoea pilosula* Franch.	*Codonopsis* Radix	*Dang shen^#^*	474 (26.87)
1. *Rheum tanguticum Maxim*. ex Balf. *2. Rheum palmatum* L. 3. *Rheum officinale* Baill.	*Rhei Radix et* Rhizoma	*Da huang*	474 (26.87)
1. *Cimicifuga heracleifolia* Kom. 2. *Actaea heracleifolia* (Kom.)	*Cimicifugae* Rhizoma	*Sheng ma*	458 (25.96)
*Gastrodia elata* Blume	*Gastrodiae* Rhizoma	*Tian ma^#^*	445 (25.23)

^*^Botanical names based on the World Flora Online (WFO) Plant List (https://wfoplantlist.org/ accessed November 6, 2023). ^#^Dietary medicinal herb defined by the official catalog published by China's National Health Commission in 2020 (China National Health and Family Planning Commission, 2018; China National Health Commission, 2020).

### 2.5 Data analysis

IBS SPSS statistics (version 28.0, IBM Corp., Armonk, NY, USA) was employed for the descriptive analyses of patients' characteristics and treatment information. Categorical variables were presented as frequency and percentage, while continuous variables were expressed as mean with standard deviation. Furthermore, IBS SPSS Modeler 18.0 was utilized to generate association rules between herbs and symptoms, employing the Apriori algorithm.

## 3 Results

### 3.1 Summary of the research procedure

**Figure 1:**
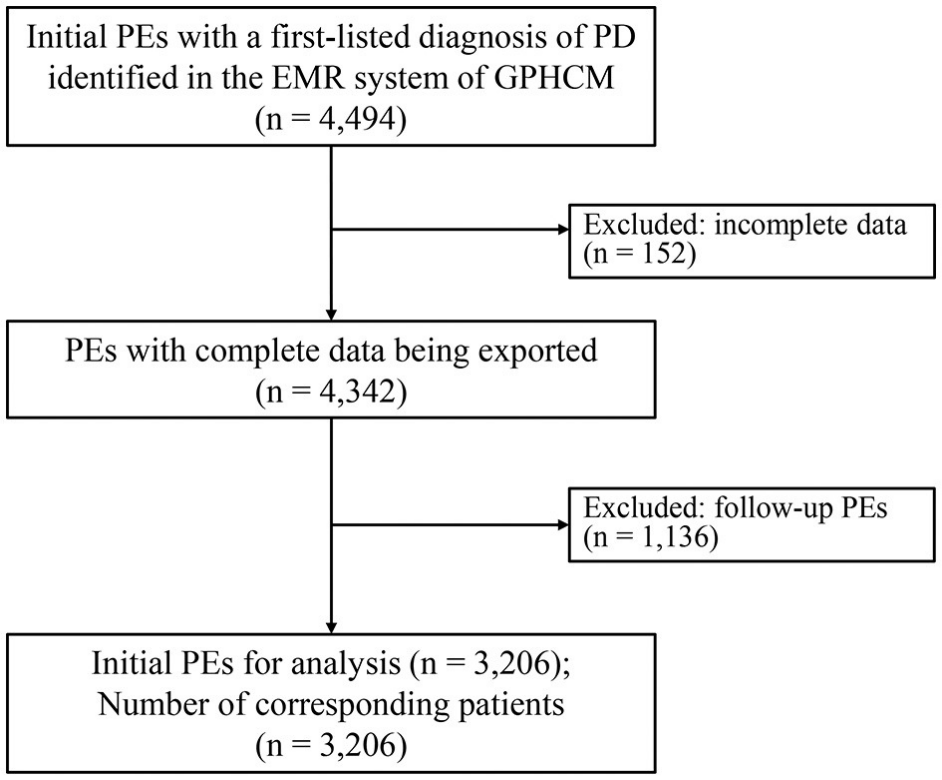
Flowchart of the study (EMRs, electronic medical records; PD, Parkinson's disease; PE, patient encounter).

A total of 4,494 outpatient initials PEs with a first-listed diagnosis of PD were identified and exported from the EMR system of GPHCM. During the screening procedure, 152 PEs were excluded for incomplete data, and 1,136 PEs were excluded because they were follow-up PEs rather than initial PEs. Ultimately, 3,206 PEs, each corresponding to an individual patient, were included in the analyses (Figure 1).

### 3.2 Clinical features of all patients

#### 3.2.1 Demographics and general characteristics

There were 3,206 patients involved in this study as each of the included PEs corresponds to one individual patient. Out of the 3,206 eligible patients with a first-listed diagnosis of PD, 1,632 (50.90%) were male. The average age of the patients was 66.04 ± 3.98 years old, with the most frequently reported onset age of parkinsonism falling within the range of 60 to 70 years (*n* = 964, 30.07%). Disease duration from the onset of motor symptoms was available form 2,548 (79.48%) of the patients. Among those with a confirmed PD diagnosis (*n* = 1,485), the mean disease duration was 4.59 ± 4.26 years, while patients with suspected PD diagnosis (*n* = 348) had an average disease duration of 1.11 ± 1.68 years. PD patients sought medical care in various outpatient departments during their initial visits to the hospital. The most common one was the internal medicine outpatient department (*n* = 2,436, 75.98%), followed by PD chronic care clinic (*n* = 444, 13.85%) (Table 2).

**Table 2:** Characteristics of patients with first-listed diagnosis of Parkinson's disease.

**Item (continuous variable)**		**Mean (SD)**
Age (years)		66.04 (3.98)
Disease duration (years, valid *n =* 2,549)^#^	Confirmed diagnosis of PD at initial visit (valid *n =* 1,485)	4.59 (4.26)
	Suspected diagnosis of PD at initial visit (valid *n =* 348)	1.11 (1.68)
	Unclear status of PD diagnosis at initial visit (valid *n =* 716)	2.60 (3.37)
**Item (categorical variable)**	**Category**	**Number (%)**
Gender	Male	1,632 (50.90)
	Female	1,574 (49.10)
Confirmed diagnosis of PD at initial visit		1,930 (60.20)
Not under regular antiparkinsonian treatment (*n =* 61, 3.16%^*^)	Waiting to initiate antiparkinsonian treatment	39 (2.02)^*^
	Intolerance to side effects	21 (1.09)^*^
	Refusal of conventional antiparkinsonian medications	11 (0.57)^*^
Under regular antiparkinsonian treatment (*n =* 1,869, 96.84%^*^)	Insufficient treatment response	77 (3.99)^*^
	Progress of PD	312 (16.17)^*^
	Non-motor symptoms	233 (12.07)^*^
	Complementary to current treatments	1,237 (64.09)^*^
Suspected diagnosis of PD at initial visit		395 (12.32)
Unclear status of PD diagnosis at initial visit		881 (27.48)
PD onset age	Unclear	471 (14.69)
	≤ 50	410 (12.79)
	>50 but ≤ 60	742 (23.14)
	>60 but ≤ 70	964 (30.07)
	>70 but ≤ 80	497 (15.50)
	>80	122 (3.81)
Outpatient departments	Internal medicine outpatient clinic	2,436 (75.98)
	PD chronic care clinic	444 (13.85)
	Acupuncture and Moxibustion department	124 (3.87)
	Surgical outpatient clinic	55 (1.72)
	Orthopedic outpatient clinic	40 (1.25)
	Emergency department	31 (0.97)
	Others	76 (2.37)

^#^Time from the onset of motor symptoms; ^*^Percentage among 1,930 PEs with confirmed PD diagnosis; PD, Parkinson's disease.

Among 1,930 patients with a confirmed diagnosis of PD, 61 (3.16%) opted not to undergo regular antiparkinsonian medications. This decision may be attributed to side effects intolerance, a deliberate choice to postpone treatment initiation at early stage, or a refusal to be prescribed conventional antiparkinsonian medications. The remaining 1,869 (96.84%) patients had adhered to regular conventional antiparkinsonian medications. Among this cohort, 1,237 (64.09%) patients sought additional Chinese medicine treatment alongside their existing treatments therapies without providing detailed reasons. Other specific reasons for seeking Chinese medicine involved insufficient treatment response to conventional medications, the “wearing off” of medications as PD progressed to advanced stages, and insufficient relief of non-motor symptoms (Table 2).

#### 3.2.2 Clinical manifestations of patients with first-listed diagnosis of PD

Rest tremor, bradykinesia, rigidity and postural instability constitute the four typical motor symptoms, either recorded individually or in various combinations by the 3,206 PEs. Among these symptoms, rest tremor emerged as the most frequently documented symptom by 1,987 (61.98%) PEs, followed by bradykinesia (*n* = 1,678, 52.34%) and rigidity (*n* = 1,305, 40.70%). Postural instability was recorded by a limited number of PEs, specifically 551 (17.19%). The proportion of rest tremor and rigidity were notably high among patients with suspected PD diagnosis according to preliminary examinations.

Motor complications were not common among the initial PEs, with only 84 (2.62%) recording motor fluctuations, and 82 (2.56%) documenting dyskinesia. Motor complications were predominantly reported among patients with a confirmed diagnosis of PD (Table 3).

**Table 3:** Clinical manifestations of patients with first-listed diagnosis of Parkinson's disease.

**Symptoms**		**All patients (*n =* 3,206)**	**Confirmed PD (total *n =* 1,930)**	**Suspected PD (total *n =* 395)**	**Unclear status of PD diagnosis (total *n =* 881)**
Motor symptoms	Rest tremor	1,987 (61.98)	1,136 (58.86)	303 (76.71)	548 (62.20)
	Bradykinesia	1,678 (52.34)	989 (51.24)	233 (58.99)	456 (51.76)
	Rigidity	1,305 (40.70)	775 (40.16)	199 (50.38)	331 (37.57)
	Postural instability	551 (17.19)	336 (17.41)	76 (19.24)	139 (15.78)
Motor complications	Motor fluctuations	84 (2.62)	83 (4.30)	0 (0)	1 (0.11)
	Dyskinesia	82 (2.56)	78 (4.04)	0 (0)	4 (0.45)
Non-motor symptoms	Constipation	1,022 (31.88)	748 (38.76)	105 (26.58)	169 (19.18)
	Sleep disturbance	810 (25.27)	588 (30.47)	69 (17.47)	153 (17.37)
	Fatigue	703 (21.93)	448 (23.21)	78 (19.75)	177 (20.09)
	Musculoskeletal pain	559 (17.44)	368 (19.07)	63 (15.95)	128 (14.53)
	Urinary problems	287 (8.95)	216 (11.19)	23 (5.82)	48 (5.45)
	Anxiety/depression	235 (7.33)	169 (8.76)	19 (4.81)	47 (5.33)
	Cognitive impairment	202 (6.30)	110 (5.70)	43 (10.89)	49 (5.56)
	Rapid eye movement sleep behavior disorder	155 (4.83)	130 (6.74)	8 (2.03)	17 (1.93)
	Sweating	146 (4.55)	123 (6.37)	5 (1.27)	18 (2.04)
	Salivation	133 (4.15)	78 (4.04)	25 (6.33)	30 (3.41)
	Hallucinations and delusions	65 (2.03)	60 (3.11)	3 (0.76)	2 (0.23)
	Daytime sleepiness	56 (1.75)	48 (2.49)	2 (0.51)	6 (0.68)
	Swallowing dysfunction	51 (1.59)	38 (1.97)	4 (1.01)	9 (1.82)
	Restless legs	4 (0.12)	4 (0.21)	0 (0)	0 (0)

PD, Parkinson's disease.

In this study, a total of 15 non-motor symptoms were analyzed, with four of them recorded by over 10% of the PEs. These symptoms included constipation (*n* = 1,022, 31.88%), sleep disturbance (*n* = 810, 25.27%), fatigue (*n* = 703, 21.93%) and musculoskeletal pain (*n* = 559, 17.44%). Notably, these non-motor symptoms were reported not only by patients with a confirmed PD diagnosis, but also by those with suspected or unclear PD diagnosis (Table 3).

### 3.3 Treatments

Antiparkinsonian medications are recommended for managing Parkinsonism or assisting in the establishment or differentiation of a PD diagnosis based on patients' responses to medications (Postuma et al., 2015). Additionally, Chinese medicine is also recommended for Parkinsonism following the principle of syndrome differentiation (Yun and Liu, 2022). The regularity of treatment, both conventional and Chinese medicine, was conducted based on the included PEs, regardless of the status of PD diagnosis.

#### 3.3.1 Treatment categories

Among the 3,206 patients, 426 patients underwent examinations without receiving treatments. Antiparkinsonian medications were prescribed for 1,778 (55.46%) patients, either as a standalone treatment (*n* = 523, 16.31%) or in combination with CHM and/or acupuncture (*n* = 1,255, 39.15%). CHM was prescribed for 2,191 (68.34%) patients, either as a standalone treatment (*n* = 870, 27.14%) or in conjunction with antiparkinsonian medications and/or acupuncture (*n* = 1,321, 41.20%). Notably, CHM decoction was more commonly prescribed than PCHMPs (1,763 vs. 821). Acupuncture was limited in use, being administrated to only 148 PD patients. Integration of CHM and antiparkinsonian medications were the most common treatment category among patients with motor symptoms (39.99%) and non-motor symptoms (40.24%), while single use of CHM was the most common treatment for patients with motor complications (Table 4).

**Table 4:** Categories of treatment methods for Parkinson's disease.

**Categories of treatment**	**Subcategories of treatments**	**Frequency of treatment (%)**	Number of patients with motor symptoms (%, valid *n =* 2,676)^*^	Number of patients with motor complications (%, valid *n =* 159)^*^	Number of patients with non-motor symptoms (%, valid *n =* 2,055)^*^
CHM + AM +ACU	PCHMPs + AM + ACU	3 (0.09)	7 (0.26)	0 (0)	6 (0.29)
	CHM decoction + AM + ACU	5 (0.16)			
CHM + AM	PCHMPs + CHM decoction + AM	225 (7.02)	1,070 (39.99)	51 (32.08)	827 (40.24)
	PCHMPs + AM	348 (10.85)			
	CHM decoction + AM	666 (20.77)			
ACU + AM	ACU + AM	8 (0.25)	7 (0.26)	0 (0)	5 (0.24)
ACU + CHM	PCHMPs + CHM decoction + ACU	4 (0.12)	65 (2.43)	1 (0.63)	60 (2.92)
	PCHMPs + ACU	10 (0.31)			
	CHM decoction + ACU	60 (1.87)			
Only CHM	CHM decoction + PCHMPs	164 (5.12)	721 (26.94)	68 (42.77)	604 (29.39)
	Only PCHMPs	67 (2.09)			
	Only CHM decoction	639 (19.93)			
Only ACU		58 (1.81)	48 (1.79)	0 (0)	43 (2.09)
Only AM		523 (16.31)	415 (15.51)	25 (15.72)	275 (13.38)
No treatment		426 (13.29)	343 (12.82)	14 (8.81)	235 (11.44)

^*^Percentage within column; ACU, acupuncture; AM, antiparkinsonian medication; CHM, Chinese herbal medicine; PCHMP, patented Chinese herbal medicine product.

#### 3.3.2 Frequency analysis of herbs

Among the 1,764 PEs with prescriptions of CHM decoctions, the most frequently prescribed herb is *Glycyrrhiza uralensis* Fisch. (*gan cao*) (*n* = 1,252), followed by *Astragalus mongholicus* Bunge (*huang qi*) (*n* = 953), *Atractylodes macrocephala* Koidz. (*bai zhu*) (*n* = 948), *Angelica sinensis* (Oliv.) Diels (*dang gui*) (*n* = 844), and *Rehmannia glutinosa* (Gaertn.) DC. (*di huang*) (*n* = 753). It is noteworthy that these top frequently used herbs are also categorized as dietary medicinal herbs according to China National Health and Family Planning Commission (2018) and China National Health Commission (2020) (Table 1).

#### 3.3.3 Associations rules between symptoms and herbs

Association rules were generated to unveil potential connections between PD symptoms and herbs, utilizing the Apriori algorithm. Three parameters namely support, confidence and lift are presented in the association rules. Support is the prevalence of antecedent and its minimum threshold is usually predefined to avoid occasional co-occurrence (Agrawal et al., 1993; Xiong, 2021). Confidence reflects the possibility of co-occurrences of consequent and antecedent in the datasets consisting of antecedent, while lift is a value that represents the likelihood of an increase in the consequent given a particular antecedent (Han et al., 2011; Lu et al., 2020). Throughout this process, *Codonopsis pilosula* Nannf. (*dang shen*), *Panax ginseng* C. A. Mey. (*ren shen*/*hong shen*) were grouped as one type due to their similar functions in Chinese medicine theory (Zhong, 2016). As indicated in Table 5, the antecedent symptom of RBD was associated with the consequent use of *Rheum tanguticum Maxim*. ex Balf. (*da huang*) (lift = 2.28), *Codonopsis pilosula* Nannf. (*dang shen*)/*Panax ginseng* C. A. Mey. (*ren shen*/*hong shen*) (lift = 1.29), *Astragalus mongholicus* Bunge (*huang qi*) (lift = 1.28), and *Angelica sinensis* (Oliv.) Diels (*dang gui*) (lift = 1.26). Motor fluctuations were associated with *Codonopsis pilosula* Nannf. (*dang shen*)/*Panax ginseng* C. A. Mey. (*ren shen*/*hong shen*) (lift = 1.49), *Astragalus mongholicus* Bunge (*huang qi*) (lift = 1.40), and *Angelica sinensis* (Oliv.) Diels (*dang gui*) (lift = 1.28). Dyskinesia increased the use of *Angelica sinensis* (Oliv.) Diels (*dang gui*) (lift = 1.34) and *Codonopsis pilosula* Nannf. (*dang shen*)/*Panax ginseng* C. A. Mey. (*ren shen*/*hong shen*) (lift = 1.32). Unfortunately, association rules were not successfully constructed for other non-motor symptoms.

**Table 5:** Potentially effective herbs for specific symptoms based on association rules.

**Consequent**	**Antecedent**	**Support %**	**Confidence %**	Lift^*^	**No of subjects with antecedent symptom receiving consequent herb**
*Da huang*	RBD	6.69	61.02	2.28	72
*Dang shen*/*ren shen*/*hong shen*	Motor fluctuations	3.63	81.25	1.49	52
*Huang qi*	Motor fluctuations	3.63	75	1.40	48
*Dang gui*	Dyskinesia	3.29	63.79	1.34	37
*Dang shen*/*ren shen*/*hong shen*	Dyskinesia	3.29	72.41	1.32	42
*Dang shen*/*ren shen*/*hong shen*	RBD	6.69	70.34	1.29	83
*Dang gui*	Motor fluctuations	3.63	60.94	1.28	39
*Huang qi*	RBD	6.69	68.64	1.28	81
*Dang gui*	RBD	6.69	60.17	1.26	71

^*^A higher lift represents a higher likelihood of an increase in the consequent given a particular antecedent. RBD, rapid eye movement sleep behavior disorder; Association rules higher than 1.2 were presented.

#### 3.3.4 Frequency analysis of patented Chinese herbal medicine products

Patented Chinese herbal medicine products (PCHMPs) with a frequency exceeding 25 are detailed in Table 6. These PCHMPs were predominantly formulated for neurological conditions including stroke, headaches, coronary heart disease, etc., commonly observed among aged patients (State Pharmacopoeia Committee of China, 2020). Alternatively, they were targeted non-motor symptoms of PD, such as constipation and musculoskeletal pain.

**Table 6:** Frequently used patented Chinese herbal medicine products.

**Names of PCHMP**	**Frequency**	**Targeted conditions**	**Herb ingredients in *pin yin***
*Tian dan tong luo* capsule	141	Cerebral infarction	*Chuan xiong, xi qian cao, dan shen, shui zhi, tian ma, huai hua, shi chang pu, niu huang, huang qi, niu xi*
*Er shi wu wei shan hu* capsule	102	Neurological conditions such as headache, epilepsy, and neuropathic pain	*Shan hu, zhen zhu, qing jin shi, zhen zhu mu, he zi, mu xiang, hong hua, ding xiang, chen xiang, zhu sha, long gu, lu gan shi, nao shi, ci shi, yu liang tu, zhi ma, hu lu, zi wan hua, zhang ya cao, zang chang pu, bang na, da jian ju, gan cao, xi hong hua, she xiang*
*Tong fu xing shen* capsule^*^	101	Stroke with constipation	*Niu huang, tian zhu huang*
*Za chong shi san wei* pill	73	Neurological conditions such as stroke, headache, and neuropathic pain	*He zi, zhi cao wu, shi chang pu, mu xiang, she xiang, shan hu, zhen zhu, ding xiang, rou dou kou, chen xiang, yu liang tu, ci shi, gan cao*
*Nao an di* pill^*^	66	Cerebral infarction	*Chuan xiong, dang gui, hong hua, ren shen, bing pian*
*Yin xing mi huan* oral solution^*^	46	Coronary heart disease and ischemic cerebrovascular disease	Ginkgo biloba extract, tian ma extract
*Song ling xue mai kang* capsule	45	Headache, dizziness, irritability, palpitations, insomnia; hypertension, and primary hyperlipidaemia	*Song ye, ge gen, zhen zhu ceng fen*
*Zao ren an shen* capsule	36	Insomnia, memory loss, and dizziness	*Suan zao ren, dan shen, wu wei zi*
*Tian zhi* granule	34	Stroke and mild to moderate vascular dementia	*Tian ma, gou teng, shi jue ming, du zhong, sang ji sheng, fu shen, shou wu teng, huai hua, zhi zi, huang qin, niu xi, yi mu cao*
*Tian shu* tablet	31	Headache	*Chuan xiong, tian ma*
*Zhi bai di huang* pill	27	Symptoms such as sweating, hot flush, with syndrome of *yin* deficiency and *yang* uprising	*Zhi mu, huang bai, shu di huang, shan zhu yu, mu dan pi, shan yao, fu ling, ze xie*
*Yin xing tong zhi* dropping pill^*^	27	Dizziness, coronary heart disease, and angina pectoris	Ginkgo biloba extract

Functions and herb ingredients were summarized from Chinese Pharmacopeia 2020; ^*^Functions and herb ingredients from medicine instructions.

#### 3.3.5 Frequency analysis of antiparkinsonian medications

Among the initial PEs for patients with a first-listed diagnosis of PD, the most frequent prescribed antiparkinsonian medication is Levodopa (*n* = 1,450), followed by Dopaminergic agonist (*n* = 831), MAO-B inhibitors (*n* = 145), COMT inhibitors (*n* = 120), Amantadine (*n* = 32), and Anticholinergics (*n* = 30).

## 4 Discussion

### 4.1 Summary of results

Based on the analysis of 3,206 real-world EMRs, our study not only synthesized first-hand clinical expertise in prescribing CHM for PD, but also identified patient' characteristics and treatment categories. In summary, our study contributes to evidence-based Chinese medicine practice for PD, encompassing dimensions of clinical expertise, patients' preferences and values (Sackett, 1997; Dawes et al., 2005; Yates, 2013).

In the examinations of 348 initial PEs with a suspected PD diagnosis, the duration from the onset of motor symptoms to the first medical consultation was found to be 1.11 years, aligning with the average duration of 15 months reported in a previous survey in China (Wan et al., 2019). However, the majority of patients visited GPHCM after receiving a confirmed PD diagnosis, with an average delay of 4.59 years from the onset of motor symptoms. Within this group, some patients may have been following a routine of conventional antiparkinsonian medications without concurrent Chinese medicine treatment, while others may have previously undergone Chinese medicine treatments elsewhere. At this stage, ~50% of patients may have already developed motor complications (Bhidayasiri and Truong, 2008; Kim et al., 2020). Despite this, the percentage of motor complications based on the included real-world PEs was merely around 2.5%. This discrepancy may be attributed to the limited awareness of motor complications among non-PD specialists from the internal medicine outpatient clinic, which constitutes over 75% of PD-related outpatient visits. There is a need for education targeting non-PD specialists to enhance their understanding of motor complications and promote optimal management of motor complications in the later stages.

Before visiting the studied Chinese medicine hospital, 96.84% of patients adhered to regular conventional antiparkinsonian medications, whereas a mere 3.16% deviated from regular treatments. This smaller percentage comprised individuals either awaiting the initiation of antiparkinsonian treatments or those unable to tolerate the associated side effects. The delayed commencement of antiparkinsonian treatment until disease progression is not uncommon among PD patients (Stocchi et al., 2015). Additionally, intolerable side effects of antiparkinsonian medications were frequently reported (Rascol et al., 2003). Among those consistently on regular treatments, the decision to seek Chinese medicine may be influenced by factors such as a suboptimal response to conventional antiparkinsonian medications, the “wearing off” phenomenon as PD advances to later stages, and insufficient managements for non-motor symptoms.

Early diagnosis and intervention of non-levodopa for PD patients have been recommended (Pan et al., 2015; Tinelli et al., 2016), despite ongoing controversies regarding the timing and strategies for the initial pharmacological therapy for PD (Waller et al., 2021). Factors contributing to the diagnostic delay in PD may include physicians' unfamiliarity with PD symptomology (Wan et al., 2019). Given the substantial involvement of non-PD specialists, it may be imperative to educate clinicians on PD knowledge as a strategy to reduce diagnostic latency. In term of treatment, initiating non-levodopa medications early not only alleviates troublesome PD symptoms but also delays the initiation of levodopa and its subsequent side effects, potentially slowing disease progression (Murman, 2012). CHM has demonstrated potential as an effective treatment in conjunction with conventional antiparkinsonian medications in improving motor symptoms, non-motor symptoms and quality of life (Li et al., 2016; Chen et al., 2020; Liu et al., 2020a; Gu et al., 2023; Jun et al., 2023) (Supplementary File 1). However, the current study indicated that most PD patients initiated their Chinese medicine intervention from GPHCM after 4.59 years from the onset of motor symptoms. To complement early intervention in PD and enhance prognosis, there is a need for community education and promotion regarding the effectiveness of Chinese medicine interventions, alongside efforts to improve the availability and accessibility of CHM.

Rest tremor is recorded as the most frequently experienced motor symptom by over 60% of patients with either a confirmed or suspected PD diagnosis, given its widely recognized association with PD (Baumann, 2012). In contrast, postural instability is documented by < 20% of PEs, a prevalence consistent with previous report (16%) (Appeadu and Gupta, 2023). Constipation, sleep disturbance, fatigue and musculoskeletal pain are the prominent non-motor symptoms documented in the EMRs, irrespective of the diagnosis status. Their prevalence aligns with previously documented non-motor symptoms (Tanveer et al., 2018; Kwok et al., 2021; Li L. C. et al., 2021). However, other non-motor symptoms such as cognitive impairment, restless legs, and daytime sleepiness in our study were not as prevalent as reported among middle-late-stage PD patients in other studies (Kwok et al., 2021; Li L. C. et al., 2021). Non-motor symptoms like constipation, insomnia, anxiety, and depression have been reported to exert the greatest negative impact on the quality of life among PD patients (Duncan et al., 2014). In the studied Chinese medicine hospital, PCHMPs were tailored to address these commonly reported non-motor symptoms. For instance, *Zao ren an shen* capsule can be prescribed for patients experiencing sleep disturbance (Birling et al., 2022), and *Tong fu xing shen* capsule is known for its efficacy in addressing constipation among PD patients (Huang, 2012). Additionally, herb ingredients of CHM decoctions can also be modified to target specific non-motor symptoms. For example, *Rehmannia glutinosa* (Gaertn.) DC. (*da huang*) was frequently prescribed for constipation.

In the studied Chinese medicine hospital, integrated Chinese and conventional medicine emerged as the most popular treatment method, followed by the single use of CHM and single use of antiparkinsonian medications. The use of acupuncture for PD was limited, and its utilization regularity was not analyzed due to the insufficient available information. As the predominant treatment for PD, CHM decoctions were analyzed in depth.

Compared to previously published data-mining studies for PD based on Health Insurance Research Database in Taiwan Province of China (Chen et al., 2018; Lin et al., 2021), *Gastrodia elata* Blume (*tian ma*) and *Rehmannia glutinosa* (Gaertn.) DC. (*da huang*) maintained consistency in popularity among CHM prescriptions. However, the high frequency of tonifying herbs like *Astragalus mongholicus* Bunge (*huang qi*), *Atractylodes macrocephala* Koidz. (*bai zhu*), *Angelica sinensis* (Oliv.) Diels (*dang gui*), *Panax ginseng* C. A. Mey. (*ren shen*), etc. was seldom reported in the Taiwan studies (Chen et al., 2018; Lin et al., 2021). This disparity may be attributed to the use of different datasets with geographical differences. Nonetheless, tonifying herbs were frequently used for PD, as indicated by a literature review (Gu and Yuan, 2023).

Eight out of the top 15 most frequently used herbs in our study, including *Panax ginseng* C. A. Mey. (*ren shen*), *Astragalus mongholicus* Bunge (*huang qi*), *Atractylodes macrocephala* Koidz. (*bai zhu*), *Citrus aurantium* L. (*chen pi*), *Angelica sinensis* (Oliv.) Diels (*dang gui*), *Cimicifuga heracleifolia* Kom. (*sheng ma*), *Bupleurum chinense* DC. (*chai hu*) and *Glycyrrhiza uralensis* Fisch. (*gan cao*), are the herb ingredients of *Bu zhong yi qi tang* (BZYQT), a classical formula widely employed for neurodegenerative conditions such as Alzheimer's Disease and amyotrophic lateral sclerosis (Lim et al., 2018; Yang, 2023). BZYQT has been recommended by clinical guidelines for PD and PD-associated autonomous neurofunctional disorders (Luo et al., 2021; Zhao and Liu, 2021). Additionally, the logical combination of other herbs, including *Paeonia lactiflora* Pall. (*bai shao*), *Angelica sinensis* (Oliv.) Diels (*dang gui*), *Citrus aurantium* L. (*chen pi*), *Panax ginseng* C. A. Mey. (*ren shen*), *Atractylodes macrocephala* Koidz. (*bai zhu*), *Astragalus mongholicus* Bunge (*huang qi*) and *Poria cocos* (Schw.) Wolf (*fu ling*), contributes to the main ingredients of *Ren shen yang rong tang* (RSYRT). RSYRT was recommended for motor complications of PD (Liu et al., 2020). However, both classical formulas were not mentioned in other Chinese medicine clinical guidelines for PD (Wu et al., 2020; Li W. et al., 2021; Yang et al., 2021; Yun and Liu, 2022).

It is noteworthy that motor fluctuations and dyskinesia were likely to be managed using Chinese medicine herbs such as *Angelica sinensis* (Oliv.) Diels (*dang gui*), *Codonopsis pilosula* Nannf. (*dang shen*)/*Panax ginseng* C. A. Mey. (*ren shen*/*hong shen*), *Astragalus mongholicus* Bunge (*huang qi*), according to the association rules. The findings might offer a novel perspective for motor fluctuations and dyskinesia, where there is a lack of efficient treatments (Liu et al., 2020). Although the specific mechanism and effects of these herbs for motor fluctuations and dyskinesia require further examination and exploration, the findings provide a basis for future research. It is also interesting to observe that RBD was associated with prescribing *Rheum tanguticum Maxim*. ex Balf. (*da huang*), a herb specific for constipation, while RBD has been reported to be correlated with constipation (Kong et al., 2020; Chen et al., 2023). This finding indicated that PD-induced RBD may be treated via anti-constipation herbs like *Rheum tanguticum Maxim*. ex Balf. (*da huang*).

### 4.2 Mechanism of herb actions

To support the clinical utilization of the above-mentioned herbs and formulas for PD, mechanisms of herb actions for PD were summarized.

BZYQT has been widely reported to address PD-induced constipation and orthostatic hypotension (Bi et al., 2014; Chen and Wang, 2014; Wu et al., 2018). It exhibited effects in preventing reduction of tyrosine hydroxylase and accumulation of alpha-synuclein in the intestine of PD mouse model (Bi and Gao, 2015). In addition, BZYQT also exerted anti-apoptosis, anti-dementia and neuroprotective effects for ACL, Alzheimer's disease and ischemic stroke models (Lim et al., 2018; Li Q. et al., 2022; Yang, 2023).

The adjunct use of RSYRT showed superior effects compared to the single use of antiparkinsonian medications for PD with a Chinese medicine syndrome of Deficiency of *qi* and Blood in a RCT (Wen, 2013). Clinical trials also found RSYRT to be effective in improving fatigue symptom (Xu et al., 2020), and anti-microinflammation in haemodialysis patients (Hsiao et al., 2015). Moreover, RSYRT demonstrated anti-aging effects via improving insulin resistance in the brain (Zhao, 2023).

Mechanisms of individual herb actions for PD were summarized in Supplementary File 2. These frequently used herbs exerted evident antioxidant, neuroprotective, anti-apoptosis, anti-neuroinflammatory effects, except for *Citrus aurantium* L. [mainly involved anti-constipation effects (Yan et al., 2020; Gong et al., 2023)] and *Poria cocos* (Schw.) Wolf [possessing antidepressant and sedative-hypnotic effects (Shah et al., 2014; Huang et al., 2020; Pang et al., 2020; Chen et al., 2021; Kim et al., 2022)].

*Glycyrrhiza uralensis* Fisch. (*gan cao*) is the most frequently used herb for PD. A RCT indicated that 6-weeks *licorice* intake significantly improved PD symptoms without serious adverse events (Petramfar et al., 2020). Water extracts of *Glycyrrhiza uralensis* Fisch. (*gan cao*) demonstrated neuroprotective effects via regulating ERK-1/2 pathways and the mTORC1-AMPK1 axis, as well as inhibiting MAO-2 action in *in vitro* studies (Karthikkeyan et al., 2021, 2022; Ramadan et al., 2022). Its active compounds like *Licopyranocoumarin, glycyrurol*, and *isoliquiritigenin*, exerted anti-apoptosis against oxidative stress (Hwang and Chun, 2012; Fujimaki et al., 2014).

*Astragaloside* IV and *Calycosin* are bioactive compounds of *Astragalus mongholicus* Bunge (*huang qi*), they could protect dopaminergic neuron against neuroinflammation and oxidative stress, prevent dopaminergic neurodegeneration and mitigate PD symptoms, via regulating signaling ways of TLR/NF-κb and MAPK, Nrf2, nfκb/NLRP3, JAK2/STAT3, PI3K/AKT/mtor, and p38 MAPK signaling pathways (Chan et al., 2009; Liu et al., 2017; Yang C. et al., 2019; Yang J. et al., 2019; Tan et al., 2020; Xia et al., 2020; Xu et al., 2021).

*Atractylenolide* I, *atractylenolide* III, and *atractylodin* are the main bioactive compounds of *Atractylodes macrocephala* Koidz. (*bai zhu*), they decreased microglial activation, conferred protection to dopaminergic neurons, protected dopaminergic neurons from apoptosis, inflammatory cytokines and oxidant protein, attenuated transcriptional activities of NF-κb and MAPK phosphorylation in PD mouse models or *in vitro* experiments (More and Choi, 2017a,b; Jeong et al., 2019; Li H. et al., 2022).

*N-Butylidenephthalide* is a bioactive compound extracted from *Angelica sinensis* (Oliv.) Diels (*dang gui*), it can improve PD recovery efficiency in a PD mouse model (Chi et al., 2018), and block egl-1 expression to inhibit apoptosis pathways as well as raise rpn-6 expression to enhance activity of proteasomes (Fu et al., 2014).

The compound of *catalpol* is extracted from *Rehmannia glutinosa* (Gaertn.) DC. (*di huang*), and it demonstrated antioxidant, anti-inflammatory and neuroprotective effects *in vitro* (Tian et al., 2006; Bi et al., 2008a,b). Formulas consisting of *Rehmannia glutinosa* (Gaertn.) DC. (*di huang*) as a main ingredient exerted antiparkinsonian therapeutic effects via modulating apoptosis through MAPK and TLR4/NF-κb signaling ways (Tseng et al., 2014; Wang et al., 2021; He et al., 2023).

*Paeoniflorin* is one of the active compounds of *Paeonia lactiflora* Pall. (*bai shao*), it exerted neuroprotective, anti-ferroptosis, anti-neuroinflammatory, antioxidant and anti-apoptosis effects in PD mouse models and *in vitro* research, via regulating the α-synuclein/PKC-δ, Bcl-2/Bax/caspase-3, Akt/Nrf2/Gpx4, ROS/pkcδ/NF-κb, and Bcl-2/Bax signaling pathways (Sun et al., 2012; Dong et al., 2015; Zheng et al., 2016; Guo et al., 2021; Wang et al., 2022).

*Panax ginseng* C. A. Mey. (*ren shen*) extracts and compounds have been widely investigated for PD. Ginsenoside Rg1 exerted neuroprotective, anti-cytotoxicity and immunomodulatory effects in 1-Methyl-4-phenyl-1,2,3,6-tetrahydropyridine (MPTP)-induced PD mouse models. More specifically, it regulated prefrontal cortical gabaergic transmission (Liu et al., 2019), moderated the Wnt/β-catenin signaling pathway (Zhou et al., 2016), restored motor functions to physiological level, and attenuated loss of dopaminergic neurons in the substantia nigra and striatum (Jiang et al., 2015), reduced aberrant α-synuclein-mediated neuroinflammation (Heng et al., 2016). Ginsenoside Rg3 regulated glutathione cysteine ligase modulatory subunit and glutathione cysteine ligase regulatory subunit expression in rotenone-induced PD mice (Han et al., 2021) and downregulated apoptosis mediators, *egl-1* and *ced-3*, and upregulation of *sod-3* and *cat-2 in vitro* (Chalorak et al., 2021). Ginsenosides Rd and Re acted anti-apoptosis, anti-inflammatory and antioxidant effects, and maintained blood-brain barrier integrity in MPTP-induced PD mice (Choi et al., 2018a), lowered oxidative stress and neuroinflammation, induced Nrf2/heme oxygenase-1 expression and activated the dual PI3K/AKT and ERK pathways *in vitro* (Zhang et al., 2016; Qiao et al., 2022). Extract of *Panax ginseng* C. A. Mey. (*ren shen*) can also protect against dopaminergic neuronal death (Van Kampen et al., 2014; Jun et al., 2015; Ryu et al., 2018; Liu et al., 2020b), cell stress (Van Kampen et al., 2014) and mitochondrial dysfunction (Liu et al., 2020b), reduce indices of inflammation (Van Kampen et al., 2014; Ryu et al., 2018; Jeon et al., 2020), prevent apoptosis (Hu et al., 2011; Van Kampen et al., 2014), accumulation of α-synuclein aggregates (Van Kampen et al., 2014; Jeon et al., 2020) and MPTP-induced leaky gut barrier (Jeon et al., 2020), stimulate endogenous antioxidant release (Wang J. Y. et al., 2013), regulate neuronal formation and energy metabolism for survival (Kim et al., 2018). Signaling ways involved in these activities include the Bcl-2 family, the nuclear factor erythroid 2-related factor 2 pathways, NF-κB signaling pathways (Choi et al., 2018b; Jeon et al., 2021).

Extracts of *Bupleurum chinense* DC. (*chai hu*) exerted anti-inflammatory and neuroprotective effects as they can alleviate mitochondria damage in MPTP-induced PD mouse models (Jeong et al., 2018), regulate nuclear receptor-related 1 protein (Sim et al., 2017), and suppress NF-κb-mediated inflammatory pathways (Park et al., 2015).

### 4.3 Implication for clinical practice

The initial application of Chinese medicine in managing PD has been a subject of controversy. Nonetheless, Chinese medicine therapies, particularly CHM, emerges as a promising non-levodopa intervention. These therapies can be prescribed either in conjunction with antiparkinsonian medications or as standalone treatments for PD patients (Cho et al., 2018). Notably, the integration of Chinese and conventional medicine is believed to contribute significantly to improve PD symptoms (Li and Le, 2021). Our study indicated PD patients undergoing regular conventional medicine would still seek Chinese medicine to enhance their current treatments, when PD condition progressed with “wearing-off” phenomenon, or reluctance and/or intolerance to conventional treatment were observed. PD patients can also initiate their antiparkinsonian treatment with CHM, as an alternative to conventional medicine. When deciding on treatment methods, various factors should be taken into consideration, including the patients' age, individual preferences, treatment responses, tolerance of medications, the severity of PD in terms of both non-motor and motor disability, impairment in quality of life, and presence of comorbidities (Marsili et al., 2017; de Bie et al., 2020; Waller et al., 2021).

As previously discussed, each herb ingredient in BZYQT exerts one or several actions, including antioxidant, neuroprotective, anti-apoptosis, anti-neuroinflammatory, anti-constipation, antidepressant, and sedative-hypnotic effects. Furthermore, BZYQT itself exhibits potential anti-dementia and neuroprotective effects (Lim et al., 2018; Li Q. et al., 2022; Yang, 2023). We advocate for the prescription of BZYQT in the management of PD. Tailored modifications to the formula can be implemented to address specific individual non-motor symptoms, such as incorporating *Rheum tanguticum Maxim*. ex Balf. (*da huang*) for constipation. Additionally, PCHMPs can be prescribed to deal with comorbidities or accompanying symptoms. For instance, *Suan zao ren* capsule may be considered for sleep disturbances.

Moreover, many of the frequent herbs, including *Glycyrrhiza uralensis* Fisch. (*gan cao*), *Astragalus mongholicus* Bunge (*huang qi*), *Panax ginseng* C. A. Mey. (*ren shen*), etc., are categorized as dietary and herbal supplements (Coates et al., 2010; China National Health and Family Planning Commission, 2018; China National Health Commission, 2020). These can be provided as part of “food therapy” or “medicinal diet therapy” (Wu and Liang, 2018), serving as beneficial supplements in daily self-management of individuals with PD.

### 4.4 Implication for future research

Patients with PD often seek complementary therapies to improve both motor and non-motor symptoms (Ferry et al., 2002; Tan et al., 2006; Kim et al., 2009; Pecci et al., 2010; Wang Y. et al., 2013). RCT evidence has demonstrated the efficacy of CHM for PD in controlled settings with supportive findings from laboratory experiments. However, the generalizability of this evidence remains limited in nature. Real-world effects of CHM for PD remain uncertain and warrant further exploration. In addition, existing evidence focused on short-to-intermediate term effects of CHM for PD. Given the chronic and progressive nature of PD, investigating the prolonged effects and safety of long-term CHM for PD holds significant clinical value and merits thorough exploration. Furthermore, PD patients, especially those in advanced ages, often exhibit comorbidities such as Alzheimer's disease, hypertension, and others. Investigating the multi-targeted effects of CHM for these co-existing conditions is an avenue awaiting exploration.

In the present study, an association was identified between motor complications and the use of herbs such as *Angelica sinensis* (Oliv.) Diels (*dang gui*), *Astragalus mongholicus* Bunge (*huang qi*), and *Codonopsis pilosula* Nannf. (*dang shen*)/*Panax ginseng* C. A. Mey. (*ren shen*/*hong shen*). This herb combination may be utilized for motor complications in future research, and its clinical effects deserve future examination. Similarly, the exploration of treating RBD with anti-constipation herb like *Rheum tanguticum Maxim*. ex Balf. (*da huang*) is also recommended.

### 4.5 Limitations

Inevitable limitations should be acknowledged in this study. Firstly, the research relied on EMRs from a single hospital and failed to retrieve previous treatments outside the studied hospital, limiting the generalizability and reliability of the findings, despite the hospital's tertiary status and the analysis being based on data from over 3,000 patients. Secondly, the absence of recorded treatment response in the initial PEs include in the study diminishes confidence in the practical effectiveness of the concluded CHM prescriptions for PD. Prospective longitudinal studies with quantitative measurements are needed to better ascertain the regularity of “effective” CHM prescriptions for PD. Thirdly, the timing and real-world effectiveness of Chinese medicine intervention for PD remain unresolved issues that warrant further exploration.

## 5 Conclusion

The studied patients generally initiated their visits to GPHCM after receiving a PD diagnosis, typically 4.59 years after the onset of motor symptoms. These patients were commonly prescribed with CHM decoctions and PCHMPs, either as standalone treatments or in conjunction with antiparkinsonian medications. Notably, BZYQT emerged as a fundamental prescription for PD, often tailored to address both motor complications and non-motor symptoms. While previous research has demonstrated the antiparkinsonian effects of BZYQT and its individual herbal components in compound or extract forms, a pressing need exists for rigorous clinical trials to further validate and explore its effectiveness for PD and optimize its integration into the therapeutic landscape for PD.

## Data availability statement

The original contributions presented in the study are included in the article/Supplementary material, further inquiries can be directed to the corresponding authors.

## Ethics statement

The studies involving humans were approved by the Human Research Ethics Committee (HREC) of Guangdong Provincial Hospital of Chinese Medicine. The studies were conducted in accordance with the local legislation and institutional requirements. The ethics committee/institutional review board waived the requirement of written informed consent for participation from the participants or the participants' legal guardians/next of kin because The study was conducted based on electronic medical records and the identifying information of the medical records were not exported.

## Author contributions

SL: Conceptualization, Data curation, Formal analysis, Funding acquisition, Methodology, Software, Writing – original draft, Writing – review & editing. CZ: Supervision, Writing – review & editing. ZM: Data curation, Writing – review & editing. XG: Methodology, Supervision, Writing – review & editing. ZL: Investigation, Methodology, Writing – review & editing. XL: Conceptualization, Methodology, Writing – review & editing. JS: Conceptualization, Methodology, Supervision, Writing – review & editing. QS: Conceptualization, Methodology, Supervision, Writing – review & editing.

## References

[B1] Agrawal R. Imieliński T. Swami A. (1993). “Mining association rules between sets of items in large databases,” in Proceedings of the 1993 ACM SIGMOD international conference on Management of data. Washington, DC: Association for Computing Machinery, 207–216.

[B2] Appeadu M. K. Gupta V. (2023). Postural Instability. Treasure Island, FL: StatPearls Publishing. 32809741

[B3] Baumann C. R. (2012). Epidemiology, diagnosis and differential diagnosis in Parkinson's disease tremor. Parkinsonism Relat. Disord. 18, S90–S92. 10.1016/S1353-8020(National Institute for Health and Care Excellence, 2017)70029-3 22166466

[B4] Bhidayasiri R. Truong D. D. (2008). Motor complications in Parkinson disease: clinical manifestations and management. J. Neurol. Sci. 266, 204–215. 10.1016/j.jns.2007.08.028 17897677

[B5] Bi J. Jiang B. Liu J. H. Lei C. Zhang X. L. An L. J.. (2008a). Protective effects of catalpol against H_2_O_2_-induced oxidative stress in astrocytes primary cultures. Neurosci Lett. 442, 224–227. 10.1016/j.neulet.2008.07.029 18652878

[B6] Bi J. Wang X. B. Chen L. Hao S. An L. J. Jiang B.. (2008b). Catalpol protects mesencephalic neurons against MPTP induced neurotoxicity via attenuation of mitochondrial dysfunction and MAO-B activity. Toxicol In Vitro. 22, 1883–1889. 10.1016/j.tiv.2008.09.007 18840519

[B7] Bi S. Gao H. (2015). Influence of selegiline combined with center-supplementing and qi-boosting decoction on the expression of TH and α-syn in rats model with Parkinson's disease [司来吉兰联合补中益气汤对帕金森病模型大鼠结肠 TH,α-Syn 表达的影响]. J. Henan Univ. Chin. Med. 35, 512–514. 10.16367/j.issn.1003-5028.2015.03.0218

[B8] Bi S. Liu B. Gao H. (2014). Clinical observation on Bu-Zhong Yi-Qi decoction combined with selegiline for Parkinson's constipation [补中益气汤加减联合司来吉兰治疗帕金森病伴功能性便秘的疗效观察]. Guiding J. Trad. Chin. Med. Pharmacol. 20, 25–27. 10.13862/j.cnki.cn43-1446/r.2014.11.009

[B9] Birling Y. Zhu X. Avard N. Tannous C. Fahey P. P. Sarris J.. (2022). Zao Ren An Shen capsule for insomnia: a double-blind, randomized, placebo-controlled trial. Sleep. 45:zsab266. 10.1093/sleep/zsab266 34788454

[B10] Black N. (1996). Why we need observational studies to evaluate the effectiveness of health care. BMJ 312, 1215–1218. 10.1136/bmj.312.7040.1215 8634569 PMC2350940

[B11] CAER Inc (2023). Inpatient and Outpatient Coding 2023. Available online at: https://www.isbe.net/CTEDocuments/HST-L630185.pdf (accessed December 21, 2023).

[B12] Carroll V. Rossiter R. Blanchard D. (2021). Non-motor symptoms of Parkinson's disease. Austr. J. Gen. Prac. 50, 812–817. 10.31128/AJGP-07-21-6093 34713279

[B13] Chalorak P. Sanguanphun T. Limboonreung T. Meemon K. (2021). Neurorescue effects of frondoside A and ginsenoside Rg3 in *C. elegans* model of Parkinson's disease. Molecules 26:4843. 10.3390/molecules26164843 34443430 PMC8402114

[B14] Chan W. S. Durairajan S. S. Lu J. H. Wang Y. Xie L. X. Kum W. F.. (2009). Neuroprotective effects of Astragaloside IV in 6-hydroxydopamine-treated primary nigral cell culture. Neurochem. Int. 55, 414–422. 10.1016/j.neuint.2009.04.012 19409437

[B15] Chaudhuri K. R. Martinez-Martin P. Brown R. G. Sethi K. Stocchi F. Odin P.. (2007). The metric properties of a novel non-motor symptoms scale for Parkinson's disease: results from an international pilot study. Mov. Disord. 22, 1901–1911. 10.1002/mds.21596 17674410

[B16] Chen F. Chen S. Si A. Luo Y. Hu W. Zhang Y. Ma J. (2022). The long-term trend of Parkinson's disease incidence and mortality in China and a Bayesian projection from 2020 to 2030. Front. Aging Neurosci. 14:973310. 10.3389/fnagi.2022.973310 36185486 PMC9520003

[B17] Chen K. Y. Wu M. Y. Yang P. S. Chiang J. H. Hsu C. Y. Chen C. Y. Yen H. R. (2018). Utilization of Chinese herbal medicine and its association with the risk of fracture in patients with Parkinson's disease in Taiwan. J. Ethnopharmacol. 226, 168–175. 10.1016/j.jep.2018.08.021 30118835

[B18] Chen K. Z. Chen S. Ren J. Y. Lin S. Xiao M. J. Cheng L.. (2021). Antidepressant effect of acidic polysaccharides from Poria and their regulation of neurotransmitters and NLRP3 pathway. Zhongguo Zhong Yao Za Zhi 46, 5088–5095. 10.19540/j.cnki.cjcmm.20210610.705 34738405

[B19] Chen M. Wang W. (2014). Effectiveness of Buzhong Yiqi decoction for constipation in patients with Parkinson's disease [补中益气汤加减治疗帕金森病患者便秘症状的临床观察]. J. Cardio-cerebrovascular Dis. Integr. Chin. Western Med. 12, 59–60. 10.3969/j.issn.1672-1349.2014.01.0033

[B20] Chen S. Y. Xiao S. J. Lin Y. N. Li X. Y. Xu Q. Yang S. S.. (2020). Clinical efficacy and transcriptomic analysis of congrong shujing granules () in patients with Parkinson's disease and syndrome of Shen (Kidney) essence deficiency. Chin. J. Integr. Med. 26, 412–419. 10.1007/s11655-020-3080-0 32291608

[B21] Chen Y. Xu Q. Wu L. Zhou M. Lin Y. Jiang Y.. (2023). REM sleep behavior disorder correlates with constipation in de novo Chinese Parkinson's disease patients. Neurol. Sci. 44, 191–197. 10.1007/s10072-022-06381-5 36098886

[B22] Chi K. Fu R. H. Huang Y. C. Chen S. Y. Hsu C. J. Lin S. Z.. (2018). Adipose-derived stem cells stimulated with n-Butylidenephthalide exhibit therapeutic effects in a mouse model of Parkinson's disease. Cell Transplant. 27, 456–470. 10.1177/0963689718757408 29756519 PMC6038049

[B23] China National Health Commission (2020). China Official Catalogue of Dietary Medicinal Herbs. Beijing: China National Health Commission and China State Administration for Market Regulation.

[B24] China National Health and Family Planning Commission (2018). China Official Catalogue of Dietary Medicinal Herbs. Beijing: China National Health and Family Planning Commission.

[B25] Cho K. H. Kim T. H. Kwon S. Jung W. S. Moon S. K. Ko C. N.. (2018). Complementary and alternative medicine for idiopathic Parkinson's disease: an evidence-based clinical practice guideline. Front. Aging Neurosci. 10:323. 10.3389/fnagi.2018.00323 30374299 PMC6196228

[B26] Choi J. H. Jang M. Nah S. Y. Oh S. Cho I. H. (2018a). Multitarget effects of Korean Red Ginseng in animal model of Parkinson's disease: antiapoptosis, antioxidant, antiinflammation, and maintenance of blood-brain barrier integrity. J. Ginseng. Res. 42, 379–388. 10.1016/j.jgr.2018.01.002 29983619 PMC6026382

[B27] Choi J. H. Jang M. Oh S. Nah S. Y. Cho I. H. (2018b). Multi-target protective effects of gintonin in 1-methyl-4-phenyl-1,2,3,6-tetrahydropyridine-mediated model of Parkinson's disease via lysophosphatidic acid receptors. Front. Pharmacol. 9:515. 10.3389/fphar.2018.00515 29875659 PMC5974039

[B28] Coates P. M. Betz J. M. Blackman M. R. Cragg G. M. Levine M. Moss J.. (2010). Encyclopedia of Dietary Supplements, 2nd Edn. New York, NY: Taylor and Francis Group.

[B29] Dawes M. Summerskill W. Glasziou P. Cartabellotta A. Martin J. Hopayian K.. (2005). Sicily statement on evidence-based practice. BMC Med. Educ. 5, 1. 10.1186/1472-6920-5-1 15634359 PMC544887

[B30] de Bie R. M. A. Clarke C. E. Espay A. J. Fox S. H. Lang A. E. (2020). Initiation of pharmacological therapy in Parkinson's disease: when, why, and how. Lancet Neurol. 19, 452–461. 10.1016/S1474-442230036-3 32171387

[B31] Dewey R. B. Jr. (2004). Management of motor complications in Parkinson's disease. Neurology 62, S3–S7. 10.1212/WNL.62.6_suppl_4.S3 15037664

[B32] Dong H. Li R. Yu C. Xu T. Zhang X. Dong M.. (2015). Paeoniflorin inhibition of 6-hydroxydopamine-induced apoptosis in PC12 cells via suppressing reactive oxygen species-mediated PKCδ/NF-κB pathway. Neuroscience 285, 70–80. 10.1016/j.neuroscience.2014.11.008 25446358

[B33] Dreyer N. A. (2022). Strengthening evidence-based medicine with real-world evidence. The Lancet Healthy Long. 3, e641–e642. 10.1016/S2666-7568(22)00214-8 36150401

[B34] Duncan G. W. Khoo T. K. Yarnall A. J. O'Brien J. T. Coleman S. Y. Brooks D. J.. (2014). Health-related quality of life in early Parkinson's disease: the impact of nonmotor symptoms. Mov. Disord. 29, 195–202. 10.1002/mds.25664 24123307

[B35] Ferry P. Johnson M. Wallis P. (2002). Use of complementary therapies and non-prescribed medication in patients with Parkinson's disease. Postgrad. Med. J. 78, 612–614. 10.1136/pmj.78.924.612 12415085 PMC1742510

[B36] Freitas M. E. Hess C. W. Fox S. H. (2017). Motor complications of dopaminergic medications in Parkinson's disease. Semin. Neurol. 37, 147–157. 10.1055/s-0037-1602423 28511255 PMC5990008

[B37] Frucht S. J. (2004). Parkinson disease: an update. Neurologist 10, 185–194. 10.1097/01.nrl.0000131146.08278.a5 15245584 PMC4119608

[B38] Fu R. H. Harn H. J. Liu S. P. Chen C. S. Chang W. L. Chen Y. M.. (2014). n-butylidenephthalide protects against dopaminergic neuron degeneration and α-synuclein accumulation in *Caenorhabditis elegans* models of Parkinson's disease. PLoS ONE 9:e85305. 10.1371/journal.pone.0085305 24416384 PMC3885701

[B39] Fujimaki T. Saiki S. Tashiro E. Yamada D. Kitagawa M. Hattori N.. (2014). Identification of licopyranocoumarin and glycyrurol from herbal medicines as neuroprotective compounds for Parkinson's disease. PLoS ONE 9:e100395. 10.1371/journal.pone.0100395 24960051 PMC4069009

[B40] GBD 2016 Parkinson's Disease Collaborators (2018). Global, regional, and national burden of Parkinson's disease, 1990-2016: a systematic analysis for the Global Burden of Disease Study 2016. Lancet Neurol. 17, 939–953. 10.1016/S1474-4422(18)30295-3 30287051 PMC6191528

[B41] Gong Y. Liang X. Dai Y. Huang X. Su Q. Ma Y.. (2023). Prokinetic effects of *Citrus reticulata* and *Citrus aurantium* extract with/without Bupleurum chinense using multistress-induced delayed gastric emptying models. Pharm. Biol. 61, 345–355. 10.1080/13880209.2023.2173249 36728913 PMC9897790

[B42] Green L. W. Glasgow R. E. (2006). Evaluating the relevance, generalization, and applicability of research:issues in external validation and translation methodology. Eval. Health Prof. 29, 126–153. 10.1177/0163278705284445 16510882

[B43] Grimes D. Fitzpatrick M. Gordon J. Miyasaki J. Fon E. A. Schlossmacher M.. (2019). Canadian guideline for Parkinson disease. Can. Med. Assoc. J. 191, E989–E1004. 10.1503/cmaj.181504 31501181 PMC6733687

[B44] Gu C. Yuan C. (2023). TCM syndrome distribution and rule of medication in Parkinson's disease [帕金森病的中医证型分布和用药规律探析]. Shanghai J. Trad. Chin. Med. 47, 12–14.

[B45] Gu S. C. Shi R. Gaoag C. Yuan X. L. Wu Y. Zhang Y.. (2023). Traditional Chinese medicine pingchan granule for motor symptoms and functions in Parkinson's disease: a multicenter, randomized, double-blind, placebo-controlled study. Phytomedicine. 108, 154497. 10.1016/j.phymed.2022.154497 36283254

[B46] Guangdong Provincial Hospital of Chinese Medicine (2021). Introduction to Guangdong Provincial Hospital of Chinese Medicine. Available online at: http://www.gdhtcm.com/index.html (accessed December 21, 2023).

[B47] Guo K. Zhang Y. Li L. Zhang J. Rong H. Liu D.. (2021). Neuroprotective effect of paeoniflorin in the mouse model of Parkinson's disease through α-synuclein/protein kinase C δ subtype signaling pathway. Neuroreport 32, 1379–1387. 10.1097/WNR.0000000000001739 34718250

[B48] Han J. Pei J. Kamber M. (2011). Data mining: Concepts and Techniques. Amsterdam: Elsevier

[B49] Han Y. Wang T. Li C. Wang Z. Zhao Y. He J.. (2021). Ginsenoside Rg3 exerts a neuroprotective effect in rotenone-induced Parkinson's disease mice via its anti-oxidative properties. Eur. J. Pharmacol. 909:174413. 10.1016/j.ejphar.2021.174413 34391769

[B50] He Z. Q. Huan P. F. Wang L. He J. C. (2023). Compound dihuang granule changes gut microbiota of MPTP-induced Parkinson's Disease mice via inhibiting TLR4/NF-κB signaling. Neurochem. Res. 48, 3610–3624. 10.1007/s11064-023-04004-9 37561259 PMC10584754

[B51] Heng Y. Zhang Q. S. Mu Z. Hu J. F. Yuan Y. H. Chen N. H.. (2016). Ginsenoside Rg1 attenuates motor impairment and neuroinflammation in the MPTP-probenecid-induced parkinsonism mouse model by targeting α-synuclein abnormalities in the substantia nigra. Toxicol Lett. 243, 7–21. 10.1016/j.toxlet.2015.12.005 26723869

[B52] Hsiao P. J. Lin K. S. Chiu C. C. Chen H. W. Huang J. S. Kao S. Y.. (2015). Use of traditional Chinese medicine (Ren Shen Yang Rong Tang) against microinflammation in hemodialysis patients: an open-label trial. Compl. Ther. Med. 23, 363–371. 10.1016/j.ctim.2015.03.002 26051571

[B53] Hu S. Han R. Mak S. Han Y. (2011). Protection against 1-methyl-4-phenylpyridinium ion (MPP+)-induced apoptosis by water extract of ginseng (Panax ginseng C.A. Meyer) in SH-SY5Y cells. J. Ethnopharmacol. 135, 34–42. 10.1016/j.jep.2011.02.017 21349320

[B54] Huang Q. (2012). Effectiveness of Tongfu Xingshen Capsule for Constipation in Parkinson's Disease: A Randomised Controlled Trial [通腑醒神胶囊治疗帕金森氏病功能性便秘临床研究]. Guangzhou: Guangzhou University of Chinese Medicine.

[B55] Huang Y. J. Hsu N. Y. Lu K. H. Lin Y. E. Lin S. H. Lu Y. S.. (2020). Poria cocos water extract ameliorates the behavioral deficits induced by unpredictable chronic mild stress in rats by down-regulating inflammation. J. Ethnopharmacol. 258:112566. 10.1016/j.jep.2020.112566 31926986

[B56] Hwang C. K. Chun H. S. (2012). Isoliquiritigenin isolated from licorice *Glycyrrhiza uralensis* prevents 6-hydroxydopamine-induced apoptosis in dopaminergic neurons. Biosci. Biotechnol. Biochem. 76, 536–543. 10.1271/bbb.110842 22451397

[B57] Jeon H. Kim H. Y. Bae C. H. Lee Y. Kim S. (2020). Korean Red Ginseng regulates intestinal tight junction and inflammation in the colon of a Parkinson's disease mouse model. J. Med. Food. 23, 1231–1237. 10.1089/jmf.2019.4640 33121350

[B58] Jeon H. Kim H. Y. Bae C. H. Lee Y. Koo S. Kim S.. (2021). Korean red ginseng decreases 1-methyl-4-phenylpyridinium-induced mitophagy in SH-SY5Y cells. J. Integr. Med. 19, 537–544. 10.1016/j.joim.2021.09.005 34580047

[B59] Jeong J. S. Piao Y. Kang S. Son M. Kang Y. C. Du X. F.. (2018). Triple herbal extract DA-9805 exerts a neuroprotective effect via amelioration of mitochondrial damage in experimental models of Parkinson's disease. Sci. Rep. 8:15953. 10.1038/s41598-018-34240-x 30374025 PMC6206089

[B60] Jeong Y. H. Li W. Go Y. Oh Y. C. (2019). Atractylodis rhizoma alba attenuates neuroinflammation in BV2 microglia upon LPS stimulation by inducing HO-1 activity and inhibiting NF-κB and MAPK. Int. J. Mol. Sci. 20:4015. 10.3390/ijms20164015 31426492 PMC6720582

[B61] Jiang W. Wang Z. Jiang Y. Lu M. Li X. (2015). Ginsenoside Rg1 ameliorates motor function in an animal model of Parkinson's disease. Pharmacology 96, 25–31. 10.1159/000431100 26065578

[B62] Jun P. Zhao H. Jung I. C. Kwon O. Han C. H. Won J.. (2023). Efficacy of herbal medicine treatment based on syndrome differentiation for Parkinson's disease: a systematic review and meta-analysis of randomized placebo-controlled clinical trials. Front. Pharmacol. 14:1108407. 10.3389/fphar.2023.1108407 36925641 PMC10012343

[B63] Jun Y. L. Bae C. H. Kim D. Koo S. Kim S. (2015). Korean Red Ginseng protects dopaminergic neurons by suppressing the cleavage of p35 to p25 in a Parkinson's disease mouse model. J. Ginseng Res. 39, 148–154. 10.1016/j.jgr.2014.10.003 26045688 PMC4452523

[B64] Karthikkeyan G. Behera S. K. Upadhyay S. S. Pervaje R. Prasad T. S. K. Modi P. K.. (2022). Metabolomics analysis highlights Yashtimadhu (*Glycyrrhiza glabra* L.)-mediated neuroprotection in a rotenone-induced cellular model of Parkinson's disease by restoring the mTORC1-AMPK1 axis in autophagic regulation. Phytother. Res. 36, 2207–2222. 10.1002/ptr.7449 35307886

[B65] Karthikkeyan G. Prabhu A. Pervaje R. Pervaje S. K. Modi P. K. Prasad T. S. K.. (2021). Data on dose-dependent cytotoxicity of rotenone and neuroprotection conferred by Yashtimadhu (*Glycyrrhiza glabra* L.) in an *in vitro* Parkinson's disease model. Data Brief 39:107535. 10.1016/j.dib.2021.107535 34820486 PMC8601963

[B66] Kim D. Kwon S. Jeon H. Ryu S. Ha K. T. Kim S.. (2018). Proteomic change by Korean Red Ginseng in the substantia nigra of a Parkinson's disease mouse model. J. Ginseng Res. 42, 429–435. 10.1016/j.jgr.2017.04.008 30337802 PMC6187050

[B67] Kim H. Park I. Park K. Park S. Kim Y. I. Park B. G.. (2022). The positive effects of poria cocos extract on quality of sleep in insomnia rat models. Int. J. Environ. Res. Public Health. 19:6629. 10.3390/ijerph19116629 35682214 PMC9180690

[B68] Kim H. J. Mason S. Foltynie T. Winder-Rhodes S. Barker R. A. Williams-Gray C. H.. (2020). Motor complications in Parkinson's disease: 13-year follow-up of the CamPaIGN cohort. Mov. Disord. 35, 185–190. 10.1002/mds.27882 31965629 PMC7063985

[B69] Kim S. R. Lee T. Y. Kim M. S. Lee M. C. Chung S. J. (2009). Use of complementary and alternative medicine by Korean patients with Parkinson's disease. Clin. Neurol. Neurosurg. 111, 156–160. 10.1016/j.clineuro.2008.09.011 18977584

[B70] Kong W. L. Huang Y. Qian E. Morris M. J. (2020). Constipation and sleep behaviour disorder associate with processing speed and attention in males with Parkinson's disease over five years follow-up. Sci. Rep. 10:19014. 10.1038/s41598-020-75800-4 33149217 PMC7643116

[B71] Kwok J. Y. Y. Huang T. W. Tretriluxana J. Auyeung M. Chau P. H. Lin C. C.. (2021). Symptom burden and unmet support needs of patients with Parkinson's disease: a cross-sectional study in asia-pacific regions. J. Am. Med. Dir. Assoc. 22, 1255–1264. 10.1016/j.jamda.2020.09.012 33268298

[B72] Lee H. M. Koh S. B. (2015). Many faces of Parkinson's disease: non-motor symptoms of Parkinson's disease. J. Mov. Disord. 8, 92–97. 10.14802/jmd.15003 26090081 PMC4460545

[B73] LeWitt P. A. Chaudhuri K. R. (2020). Unmet needs in Parkinson disease: motor and non-motor. Parkinsonism Relat. Disord. 80, S7–s12. 10.1016/j.parkreldis.2020.09.024 33349582

[B74] Li D. Liu Y. Wang J. Tong Y. Mei X. (2011). Basic Theory of Traditional Chinese Medicine, 中医基础理论*, published in Chinese*. Beijing: People's Medical Publishing House.

[B75] Li H. Wang F. Zhou Z. Jiang X. Li F. Feng Y.. (2022). Atractylon, a novel dopamine 2 receptor agonist, ameliorates Parkinsonian like motor dysfunctions in MPTP-induced mice. NeuroToxicology 89, 121–126. 10.1016/j.neuro.2022.01.010 35104500

[B76] Li L. C. Chen J. Zhu X. B. Guo M. Chen Q. Fang H. M.. (2021). Trends of complications in patients with Parkinson's disease in seven major cities of China from 2016 to 2019. Int. Clin. Psychopharmacol. 36, 274–278. 10.1097/YIC.0000000000000370 34102650

[B77] Li M. Yang H. M. Luo D. X. Chen J. Z. Shi H. J. (2016). Multi-dimensional analysis on Parkinson's disease questionnaire-39 in Parkinson's patients treated with Bushen Huoxue Granule: a multicenter, randomized, double-blinded and placebo controlled trial. Compl. Ther. Med. 29, 116–120. 10.1016/j.ctim.2016.09.008 27912935

[B78] Li Q. Cao M. Wei Z. Mei J. Zhang Y. Li M.. (2022). The protective effect of Buzhong Yiqi decoction on ischemic stroke mice and the mechanism of gut microbiota. Front. Neurosci. 16:956620. 10.3389/fnins.2022.956620 36590298 PMC9798918

[B79] Li S. Le W. (2021). Parkinson's disease in traditional Chinese medicine. The Lancet Neurol. 20:262. 10.1016/S1474-442230224-8 31182383

[B80] Li W. Gan J. Liu Z. (2021). Expert consensus on integrated traditional Chinese and Western medicine management for sleep disorder in Parkinson's Disease (2021 edition) [帕金森病睡眠障碍中西医结合管理专家共识(2021)]. J. Shanghai Univ. Chin. Med. 35, 1–6.

[B81] Lim H. S. Kim Y. J. Sohn E. Yoon J. Kim B. Y. Jeong S. J.. (2018). Bojungikgi-Tang, a traditional herbal formula, exerts neuroprotective effects and ameliorates memory impairments in Alzheimer's Disease-like experimental models. Nutrients 10:1952. 10.3390/nu10121952 30544702 PMC6316759

[B82] Lin C. H. Chiu H. E. Wu S. Y. Tseng S. T. Wu T. C. Hung Y. C.. (2021). Chinese herbal products for non-motor symptoms of Parkinson's disease in Taiwan: a population-based study. Front. Pharmacol. 11:615657. 10.3389/fphar.2020.615657 33584294 PMC7873047

[B83] Liu M. Hu C. Zhang Y. Li Q. Zhang Q. Fang Y.. (2020a). Effect of Huatan Jieyu granules in treatment of Parkinson's disease patients with sleep disorder identified as symptom pattern of phlegma-heat-stirring wind. J. Tradit Chin. Med. 40, 461–466. 10.19852/j.cnki.jtcm.2020.03.015 32506861

[B84] Liu M. Yu S. Wang J. Qiao J. Liu Y. Wang S.. (2020b). Ginseng protein protects against mitochondrial dysfunction and neurodegeneration by inducing mitochondrial unfolded protein response in *Drosophila melanogaster* PINK1 model of Parkinson's disease. J. Ethnopharmacol. 247:112213. 10.1016/j.jep.2019.112213 31562951

[B85] Liu X. Zhang J. Wang S. Qiu J. Yu C. (2017). Astragaloside IV attenuates the H2O2-induced apoptosis of neuronal cells by inhibiting α-synuclein expression via the p38 MAPK pathway. Int. J. Mol. Med. 40, 1772–1780. 10.3892/ijmm.2017.3157 29039448 PMC5716437

[B86] Liu Y. Zong X. Huang J. Guan Y. Li Y. Du T.. (2019). Ginsenoside Rb1 regulates prefrontal cortical GABAergic transmission in MPTP-treated mice. Aging 11, 5008–5034. 10.18632/aging.102095 31314744 PMC6682523

[B87] Liu Z. Li W. Chen H. (2020). The expert consensus on the diagnosis and treatment of integrated traditional Chinese and Western medicine in Parkinson's disease movement complications (2020) [帕金森病运动并发症中西医结合诊治专家共识 (2020)]. Chin. J. Neuroimmunol. Neurol. 27, 247–252. 10.3969/j.issn.1006-2963.2020.04.001

[B88] Lökk J. Nilsson M. (2010). Frequency, type and factors associated with the use of complementary and alternative medicine in patients with Parkinson's disease at a neurological outpatient clinic. Parkinsonism Relat. Disord. 16, 540–544. 10.1016/j.parkreldis.2010.06.007 20655794

[B89] Lu P. H. Keng J. L. Kuo K. L. Wang Y. F. Tai Y. C. Kuo C. Y. (2020). An Apriori algorithm-based association rule analysis to identify herb combinations for treating uremic pruritus using Chinese herbal bath therapy. Evid. Based Compl. Alter. Med. 2020:8854772 10.1155/2020/8854772 33299462 PMC7704140

[B90] Luo X. Li Z. Zhu M. Xu P. Guo X. Wu Z.. (2021). Traditional Chinese medicine expert consensus on diagnosis and treatment of Parkinson's disease (Tremor and Spasm disease) [帕金森病 (颤拘病) 中医临床诊疗专家共识]. J. Trad. Chin. Med. 62, 2109–2116. 10.13288/j.11-2166/r.2021.23.017

[B91] Marsili L. Marconi R. Colosimo C. (2017). “Chapter twelve - treatment strategies in early Parkinson's disease,” in International Review of Neurobiology, eds. K. P. Bhatia, K. R. Chaudhuri, M. Stamelou (New York, NY: Academic Press), 345–360. 10.1016/bs.irn.2017.01.002 28554414

[B92] Mehndiratta M. Garg R. K. Pandey S. (2011). Nonmotor symptom complex of Parkinson's disease–an under-recognized entity. J. Assoc. Physicians India. 59, 302–308. 21751608

[B93] More S. Choi D. K. (2017a). Neuroprotective role of atractylenolide-I in an *in vitro* and *in vivo* model of Parkinson's Disease. Nutrients 9:451. 10.3390/nu9050451 28468332 PMC5452181

[B94] More S. V. Choi D. K. (2017b). Atractylenolide-I protects human SH-SY5Y Cells from 1-Methyl-4-Phenylpyridinium-induced apoptotic cell death. Int. J. Mol. Sci. 18:1012. 10.3390/ijms18051012 28481321 PMC5454925

[B95] Murman D. L. (2012). Early treatment of Parkinson's disease: opportunities for managed care. Am. J. Managed Care 18:S183. 23039867

[B96] National Institute for Health and Care Excellence (2017). Parkinson's Disease in Adults (NICE guideline NH*71)*. London: National Institute for Health and Care Excellence

[B97] National Institute of Neurological Disorders and Stroke (2004). Parkinson's Disease: Challenges, Progress, and Promise. Bethesda, MD: National Institute of Neurological Disorders and Stroke.

[B98] O'Sullivan S. S. Williams D. R. Gallagher D. A. Massey L. A. Silveira-Moriyama L. Lees A. J.. (2008). Nonmotor symptoms as presenting complaints in Parkinson's disease: a clinicopathological study. Mov. Disord. 23, 101–106. 10.1002/mds.21813 17994582

[B99] Pan W. Liu J. Chen X. Wang Q. Wu Y. Bai Y.. (2015). A practical consensus guideline for the integrative treatment of Parkinson's disease in Shanghai, China. Integr. Med. Int. 2, 56–62. 10.1159/000435813

[B100] Pang Y. Zhu S. Pei H. (2020). Pachymic acid protects against cerebral ischemia/reperfusion injury by the PI3K/Akt signaling pathway. Metab. Brain Dis. 35, 673–680. 10.1007/s11011-020-00540-3 32140824

[B101] Park W. H. Kang S. Piao Y. Pak C. J. Oh M. S. Kim J.. (2015). Ethanol extract of *Bupleurum falcatum* and saikosaponins inhibit neuroinflammation via inhibition of NF-κB. J. Ethnopharmacol. 174, 37–44. 10.1016/j.jep.2015.07.039 26231448

[B102] Parkinson's Disease and Movement Disorders Group from Neurology Branch of Chinese Medical Association and Parkinson's Disease and Movement Disorders Group from Neurology Branch of Chinese Medical Doctor Association (2020). Guideline for diagnosis and treatment of Parkinson's disease in China (the fourth edition) [中国帕金森病治疗指南(第四版)]. Chin. J. Neurol. 53:14. 10.3760/cma.j.cn113694-20200331-0023330704229

[B103] Pecci C. Rivas M. J. Moretti C. M. Raina G. Ramirez C. Z. Díaz S.. (2010). Use of complementary and alternative therapies in outpatients with Parkinson's disease in Argentina. Mov. Disord. 25, 2094–2098. 10.1002/mds.23235 20721921

[B104] Petramfar P. Hajari F. Yousefi G. Azadi S. Hamedi A. (2020). Efficacy of oral administration of licorice as an adjunct therapy on improving the symptoms of patients with Parkinson's disease, A randomized double blinded clinical trial. J. Ethnopharmacol. 247:112226. 10.1016/j.jep.2019.112226 31574343

[B105] Postuma R. B. Berg D. Stern M. Poewe W. Olanow C. W. Oertel W.. (2015). MDS clinical diagnostic criteria for Parkinson's disease. Mov. Disord. 30, 1591–1601. 10.1002/mds.26424 26474316

[B106] Pringsheim T. Day G. S. Smith D. B. Rae-Grant A. Licking N. Armstrong M. J.. (2021). Dopaminergic therapy for motor symptoms in early parkinson disease practice guideline summary. A report of the AAN guideline subcommittee. Neurology 97, 942–957. 10.1212/WNL.0000000000012868 34782410 PMC8672433

[B107] Qiao J. Zhao Y. Liu Y. Zhang S. Zhao W. Liu S.. (2022). Neuroprotective effect of Ginsenoside Re against neurotoxin-induced Parkinson's disease models via induction of Nrf2. Mol. Med. Rep. 25:12731. 10.3892/mmr.2022.12731 35543148 PMC9133950

[B108] Rajendran P. R. Thompson R. E. Reich S. G. (2001). The use of alternative therapies by patients with Parkinson's disease. Neurology. 57, 790–794. 10.1212/WNL.57.5.790 11552005

[B109] Ramadan S. Sabry M. M. Saad M. A. Angeloni S. Sabry O. M. Caprioli G.. (2022). Dismantling Parkinson's disease with herbs: MAO-B inhibitory activity and quantification of chemical constituents using HPLC-MS/MS of Egyptian local market plants. Nat. Prod. Res. 36, 5766–5771. 10.1080/14786419.2021.2013836 34894897

[B110] Rascol O. Payoux P. Ory F. Ferreira J. J. Brefel-Courbon C. Montastruc J. L.. (2003). Limitations of current Parkinson's disease therapy. Annal. Neurol. 53, S3–S15. 10.1002/ana.10513 12666094

[B111] Rukavina K. Batzu L. Boogers A. Abundes-Corona A. Bruno V. Chaudhuri K. R.. (2021). Non-motor complications in late stage Parkinson's disease: recognition, management and unmet needs. Expert Rev. Neurother. 21, 335–352. 10.1080/14737175.2021.1883428 33522312

[B112] Ryu S. Jeon H. Koo S. Kim S. (2018). Korean Red Ginseng enhances neurogenesis in the subventricular zone of 1-methyl-4-phenyl-1,2,3,6-tetrahydropyridine-treated mice. Front. Aging Neurosci. 10:355. 10.3389/fnagi.2018.00355 30459594 PMC6232267

[B113] Sackett D. L. (ed.). (1997). Evidence-Based Medicine. Seminars in Perinatology. Amsterdam: Elsevier. 10.1016/s0146-0005(97)80013-4 9190027

[B114] Sanson-Fisher R. W. Bonevski B. Green L. W. D'Este C. (2007). Limitations of the randomized controlled trial in evaluating population-based health interventions. Am. J. Prev. Med. 33, 155–161. 10.1016/j.amepre.2007.04.007 17673104

[B115] Santos García D. de Deus Fonticoba T. Suárez Castro E. Borrué C. Mata M. Solano Vila B.. (2019). Non-motor symptoms burden, mood, and gait problems are the most significant factors contributing to a poor quality of life in non-demented Parkinson's disease patients: Results from the COPPADIS Study Cohort. Parkinsonism Relat Disord. 66, 151–157. 10.1016/j.parkreldis.2019.07.031 31409572

[B116] Santos-García D. de Deus Fonticoba T. Suárez Castro E. Aneiros Díaz A. McAfee D. Catalán M. J.. (2020). Non-motor symptom burden is strongly correlated to motor complications in patients with Parkinson's disease. Eur. J. Neurol. 27, 1210–1223. 10.1111/ene.14221 32181979

[B117] Shah A. D. Quinn N. J. Chaudhry A. Sullivan R. Costello J. O'Riordan D.. (2019). Recording problems and diagnoses in clinical care: developing guidance for healthcare professionals and system designers. BMJ Health Care Inf. 26:100106. 10.1136/bmjhci-2019-100106 31874855 PMC7062352

[B118] Shah V. K. Choi J. J. Han J. Y. Lee M. K. Hong J. T. Oh K. W.. (2014). Pachymic acid enhances pentobarbital-induced sleeping behaviors via GABAA-ergic systems in mice. Biomol. Ther. 22, 314–320. 10.4062/biomolther.2014.045 25143810 PMC4131518

[B119] Sim Y. Park G. Eo H. Huh E. Gu P. S. Hong S. P.. (2017). Protective effects of a herbal extract combination of *Bupleurum falcatum, Paeonia suffruticosa*, and *Angelica dahurica* against MPTP-induced neurotoxicity via regulation of nuclear receptor-related 1 protein. Neuroscience 340, 166–175. 10.1016/j.neuroscience.2016.10.029 27771535

[B120] State Pharmacopoeia Committee of China (2020). Chinese Pharmacopoeia. Beijing: China Medical Science and Technology Press.

[B121] Stocchi F. Vacca L. Radicati F. G. (2015). How to optimize the treatment of early stage Parkinson's disease. Transl. Neurodegen. 4:4. 10.1186/2047-9158-4-4 25973179 PMC4429368

[B122] Sun R. Wang K. Wu D. Li X. Ou Y. (2012). Protective effect of paeoniflorin against glutamate-induced neurotoxicity in PC12 cells via Bcl-2/Bax signal pathway. Folia Neuropathol. 50, 270–276. 10.5114/fn.2012.30527 23023341

[B123] Tan L. C. Lau P. N. Jamora R. D. Chan E. S. (2006). Use of complementary therapies in patients with Parkinson's disease in Singapore. Mov. Disord. 21, 86–89. 10.1002/mds.20662 16108030

[B124] Tan Y. Yin L. Sun Z. Shao S. Chen W. Man X.. (2020). Astragalus polysaccharide exerts anti-Parkinson via activating the PI3K/AKT/mTOR pathway to increase cellular autophagy level *in vitro*. Int. J. Biol. Macromol. 153, 349–356. 10.1016/j.ijbiomac.2020.02.282 32112840

[B125] Tanveer K. Attique I. Sadiq W. Ahmad A. (2018). Non-motor symptoms in patients with Parkinson's disease: a cross-sectional survey. Cureus 10:e3412. 10.7759/cureus.3412 30538900 PMC6281445

[B126] Tian Y. Y. An L. J. Jiang L. Duan Y. L. Chen J. Jiang B.. (2006). Catalpol protects dopaminergic neurons from LPS-induced neurotoxicity in mesencephalic neuron-glia cultures. Life Sci. 80, 193–199. 10.1016/j.lfs.2006.09.010 17049947

[B127] Tinelli M. Kanavos P. Grimaccia F. (2016). The Value of Early Diagnosis and Treatment in Parkinson's Disease: A Literature Review of The Potential Clinical and Socioeconomic Impact of Targeting Unmet Needs in Parkinson's Disease.

[B128] Tseng Y. T. Chang F. R. Lo Y. C. (2014). The Chinese herbal formula Liuwei dihuang protects dopaminergic neurons against Parkinson's toxin through enhancing antioxidative defense and preventing apoptotic death. Phytomedicine 21, 724–733. 10.1016/j.phymed.2013.11.001 24411708

[B129] Van Kampen J. M. Baranowski D. B. Shaw C. A. Kay D. G. (2014). Panax ginseng is neuroprotective in a novel progressive model of Parkinson's disease. Exp. Gerontol. 50, 95–105. 10.1016/j.exger.2013.11.012 24316034

[B130] Waller S. Williams L. Morales-Briceño H. Fung V. (2021). The initial diagnosis and management of Parkinson's disease. Austr. J. Gen. Prac. 50, 793–800. 10.31128/AJGP-07-21-6087 34713282

[B131] Wan Y. Zhu Y. Luo Y. Han X. Li Y. Gan J.. (2019). Determinants of diagnostic latency in Chinese people with Parkinson's disease. BMC Neurol. 19:120. 10.1186/s12883-019-1323-5 31185934 PMC6558921

[B132] Wang J. Y. Yang J. Y. Wang F. Fu S. Y. Hou Y. Jiang B.. (2013). Neuroprotective effect of pseudoginsenoside-f11 on a rat model of Parkinson's disease induced by 6-hydroxydopamine. Evid. Based Complement Alternat. Med. 2013:152798. 10.1155/2013/152798 24386001 PMC3872412

[B133] Wang L. An H. Yu F. Yang J. Ding H. Bao Y.. (2022). The neuroprotective effects of paeoniflorin against MPP(+)-induced damage to dopaminergic neurons via the Akt/Nrf2/GPX4 pathway. J. Chem. Neuroanat. 122:102103. 10.1016/j.jchemneu.2022.102103 35489613

[B134] Wang L. Yang Y. F. Chen L. He Z. Q. Bi D. Y. Zhang L.. (2021). Compound dihuang granule inhibits nigrostriatal pathway apoptosis in Parkinson's disease by suppressing the JNK/AP-1 pathway. Front. Pharmacol. 12:621359. 10.3389/fphar.2021.621359 33897417 PMC8060647

[B135] Wang Y. Xie C. L. Wang W. W. Lu L. Fu D. L. Wang X. T. Zheng G. Q. (2013). Epidemiology of complementary and alternative medicine use in patients with Parkinson's disease. J. Clin. Neurosci. 20, 1062–1067. 10.1016/j.jocn.2012.10.022 23815871

[B136] Wen X. (2013). Treating 68 cases of tremble of qixue kuixu type with the Renshen Yangrong decoction [人参养荣汤治疗气血亏虚型颤证68例]. Clin. J. Chin. Med. 5, 67–69. 10.3969/j.issn.1674-7860.2013.03.039

[B137] Wu C. Zhou X. Xie M. Lin J. Gao L. (2020). Expert consensus for TCM preventive treatment of diseases: Parkinson depression and/or anxiety [中医治未病· 帕金森抑郁和/或焦虑专家共识]. Chin. J. Inf. Trad. Chin. Med. 1, 1–5.

[B138] Wu Q. Liang X. (2018). Food therapy and medical diet therapy of traditional Chinese medicine. Clin. Nutr. Exp. 18, 1–5. 10.1016/j.yclnex.2018.01.001

[B139] Wu W. Zhang Q. Zhu Y. (2018). Clinical study on Buzhong Yiqi decoction combined with midodrine hydrochloride in treatment of orthostatic hypotension in Parkinson's disease [补中益气汤联合盐酸米多君治疗帕金森病直立性低血压疗效研究]. Shaanxi J. Trad. Chin. Med. 34, 713–716.

[B140] Xia M. L. Xie X. H. Ding J. H. Du R. H. Hu G. (2020). Astragaloside IV inhibits astrocyte senescence: implication in Parkinson's disease. J. Neuroinflam. 17:105. 10.1186/s12974-020-01791-8 32252767 PMC7137443

[B141] Xiong H. (2021). Association Analysis: Basic Concepts and Algorithms. Available online at: http://www.columbiaedu/~jwp2128/Teaching~W.4721 (accessed December 21, 2023).

[B142] Xu Y. Wang X. S. Chen Y. Shi Q. Chen T. H. (2020). A phase II randomized controlled trial of renshen yangrong tang herbal extract granules for fatigue reduction in cancer survivors. J. Pain Symptom Manage. 59, 966–973. 10.1016/j.jpainsymman.2019.10.018 31668965

[B143] Xu Z. Yang D. Huang X. Huang H. (2021). Astragaloside IV protects 6-hydroxydopamine-induced SH-SY5Y cell model of Parkinson's disease via activating the JAK2/STAT3 pathway. Front. Neurosci. 15:631501. 10.3389/fnins.2021.631501 33833662 PMC8021720

[B144] Yan S. Hao M. Yang H. Sun M. Wu B. Yue Y.. (2020). Metabolomics study on the therapeutic effect of the Chinese herb pair Fructus Aurantii Immaturus and Rhizoma Atractylodis Macrocephalae in constipated rats based on UPLC-Q/TOF-MS analysis. Ann. Palliat. Med. 9, 2837–2852. 10.21037/apm-20-280 32921064

[B145] Yang C. Mo Y. Xu E. Wen H. Wei R. Li S.. (2019). Astragaloside IV ameliorates motor deficits and dopaminergic neuron degeneration via inhibiting neuroinflammation and oxidative stress in a Parkinson's disease mouse model. Int0 Immunopharmacol. 75:105651. 10.1016/j.intimp.2019.05.036 31401385

[B146] Yang E. J. (2023). Combined treatment with Bojungikgi-Tang (Buzhong Yiqi decoction) and riluzole attenuates cell death in TDP-43-expressing cells. Chin. J. Integr. Med. 10.1007/s11655-023-3557-8. [Epub ahead of print]. 37695446

[B147] Yang J. Jia M. Zhang X. Wang P. (2019). Calycosin attenuates MPTP-induced Parkinson's disease by suppressing the activation of TLR/NF-κB and MAPK pathways. Phytother. Res. 33, 309–318. 10.1002/ptr.6221 30421460

[B148] Yang N. Liu W. Ning H. Liu Z. Xu L. Zhang J.. (2021). Expert consensus on diagnosis and treatment of depression in Parkinson's disease with integrated traditional Chinese and Western medicine (2021 edition) [帕金森病抑郁中西医结合诊断与治疗专家共识 (2021 年版)]. Chin. J. Contemp. Neurol. Neurosurg. 21:1027. 10.3969/j.issn.1672-6731.2021.12.002

[B149] Yates C. (2013). Evidence-based practice: the components, history, and process. Counsel. Outcome Res. Eval. 4, 41–54. 10.1177/2150137812472193

[B150] Yun C. Liu Z. (2022). Expert consensus on integrated traditional Chinese and Western medicine in the treatment of early stage Parkinson's disease (2021) [中西医结合治疗早期帕金森病专家共识 (2021)]. Shanghai J. Trad. Chin. Med. 56, 1–6. 10.16305/j.1007-1334.2021.2110003

[B151] Zhang X. Wang Y. Ma C. Yan Y. Yang Y. Wang X.. (2016). Ginsenoside Rd and ginsenoside Re offer neuroprotection in a novel model of Parkinson's disease. Am. J. Neurodegener. Dis. 5, 52–61. 27073742 PMC4788731

[B152] Zhao J. (2023). 18F-FDG Autoradiography Combined with Insulin to Evaluate the Effect of Renshen Yangrong Decoction on Local Brain Glucose Metabolism in Aged Mice [(LeWitt and Chaudhuri, 2020)F-FDG放射自显影技术结合胰岛素评价人参养荣汤对老年小鼠大脑局部葡萄糖代谢的研究*]* [Doctral Degree]. Changchun: Jilin University (2023).

[B153] Zhao Y. Liu Z. (2021). Consensus among expers on the integration of Chinese and Western medicine for autonomous neurofunctional disorders induced by Parkinson's disease (2020) [帕金森病自主神经功能障碍中西医结合诊治专家共识 (2020)]. J. Nanjing Univ. Trad. Chin. Med. 37, 6–12. 10.14148/j.issn.1672-0482.2021.0006

[B154] Zheng M. Liu C. Fan Y. Shi D. Zhang Y. (2016). Protective effects of paeoniflorin against MPP(+)-induced neurotoxicity in PC12 Cells. Neurochem. Res. 41, 1323–1334. 10.1007/s11064-016-1834-z 27053303

[B155] Zhong G. (2016). Chinese Materia Medica [中药学*], 1st Edn*. Hong Kong: Traditional Chinese Medicine Publishing co.

[B156] Zhong Q. Q. Zhu F. (2022). Trends in prevalence cases and disability-adjusted life-years of Parkinson's disease: findings from the global burden of disease study 2019. Neuroepidemiology 56, 261–270. 10.1159/000524208 35320800

[B157] Zhou T. Zu G. Zhang X. Wang X. Li S. Gong X.. (2016). Neuroprotective effects of ginsenoside Rg1 through the Wnt/β-catenin signaling pathway in both *in vivo* and *in vitro* models of Parkinson's disease. Neuropharmacology 101, 480–489. 10.1016/j.neuropharm.2015.10.024 26525190

