# Supplementary material for: Real-world Chinese herbal medicine for Parkinson's disease: a hospital-based retrospective analysis of electronic medical records
Source: Front Aging Neurosci. 2024 May 2;16:1362948. doi: 10.3389/fnagi.2024.1362948 (PMC11096516; doi:10.3389/fnagi.2024.1362948)
Supplement: Supplementary file 1 [file Data_Sheet_1.pdf]

Supplementary file 1: Studies reporting beneficial effects of Chinese herbal medicine for Parkinson's disease

Study type	Participants	Interventions	Control	Improvements in domains of assessments			
				Motor symptom	Non motor symptoms	Motor complications	Quality of life
RCT (1)	PD patients (n = 292)	*Pingchan* granule + WM	Placebo + WM	Yes	Yes	NM	Yes
RCT (2)	PD patients with Chinese medicine syndrome of Shen essence deficiency (n = 86)	*Congrong Shujing* granules + WM	Placebo + WM	Yes	NM	NM	Yes
RCT (3)	PD patients (n = 120)	*Bushen Huoxue* Granule + WM	Placebo + WM	NM	NM	NM	Yes
RCT (4)	PD patients with sleep disorders (n = 107)	*Huatan Jieyu* granules + WM	WM	NM	NM	Yes	Not mentioned
Systematic review (5)	PD patients from thirteen RCTs (n = 843)	CHM + WM	Placebo + WM	Yes	Yes	NM	Yes

Note: CHM: Chinese herbal medicine; n= number; NM: not mentioned; PD: Parkinson's disease; RCT: randomised controlled trial; WM: western medicine.

References for Supplementary file 1:

1. Gu SC, Shi R, Gaoag C, Yuan XL, Wu Y, Zhang Y, et al. Traditional Chinese medicine Pingchan granule for motor symptoms and functions in Parkinson's disease: A multicenter, randomized, double-blind, placebo-controlled study. Phytomedicine. 2023;108:154497.

2. Chen SY, Xiao SJ, Lin YN, Li XY, Xu Q, Yang SS, et al. Clinical Efficacy and Transcriptomic Analysis of Congrong Shujing Granules () in Patients with Parkinson's Disease and Syndrome of Shen (Kidney) Essence Deficiency. Chin J Integr Med. 2020;26(6):412-9.

3. Li M, Yang HM, Luo DX, Chen JZ, Shi HJ. Multi-dimensional analysis on Parkinson's disease questionnaire-39 in Parkinson's patients treated with Bushen Huoxue Granule: A multicenter, randomized, double-blinded and placebo controlled trial. Complement Ther Med. 2016;29:116-20.

4. Liu M, Hu C, Zhang Y, Li Q, Zhang Q, Fang Y, et al. Effect of Huatan Jieyu granules in treatment of Parkinson's disease patients with sleep disorder identified as symptom pattern of phlegma-heat-stirring wind. J Tradit Chin Med. 2020;40(3):461-6.

5. Jun P, Zhao H, Jung IC, Kwon O, Han CH, Won J, Jang JH. Efficacy of herbal medicine treatment based on syndrome differentiation for Parkinson's disease: A systematic review and meta-analysis of randomized placebo-controlled clinical trials. Front Pharmacol. 2023;14:1108407.
