# Supplementary material for: Real-world Chinese herbal medicine for Parkinson's disease: a hospital-based retrospective analysis of electronic medical records
Source: Front Aging Neurosci. 2024 May 2;16:1362948. doi: 10.3389/fnagi.2024.1362948 (PMC11096516; doi:10.3389/fnagi.2024.1362948)
Supplement: Supplementary file 2 [file Data_Sheet_2.pdf]

Supplementary file 2 Potential mechanisms of frequent herbs for Parkinson's disease and associated non-motor symptoms

**Herb**	**Scientific names**	**Preparation**	**Chemical constituents**	**Subject**	**Administration**	**Bioactivity**	**Mechanism of action**
Gan cao	1. *Glycyrrhiza uralensis* Fisch.<br>2. *Glycyrrhiza inflata* Batalin<br>3. *Glycyrrhiza glabra* L.	Extracts (water)	Glabridin (specific for G. Glabra), glycyrrhizic acid, liquiritin, liquitinapioside, and licoricesaponin-G2	IMR32 cell	In-vitro	Neuroprotective effect	Preventing rotenone-induced toxicity via regulating ERK-1/2 pathways (1).
		Extracts (water)	Glabridin (specific for G. Glabra), glycyrrhizic acid, liquiritin, liquitinapioside, and licoricesaponin-G2	IMR32 cell	In-vitro	Neuroprotective effect	Preventing the dysregulation of the citric acid cycle by rotenone-induced energetic stress via regulating the mtorc1-AMPK1 axis (2).
		Extracts (water)	Phenolic acids and their derivatives (vanillic acid, 5-caffeoylquinic acid, syringic acid, and p-coumaric acid)	Cell	In-vitro	Inhibiting MAO-B	Excreting the most potent MAO-B inhibitory action (3).
		Compounds	Licopyranocoumarin and glycyrurol	PC12D cell	In vitro	Neuroprotective effect	Suppressing reactive oxygen species generation, thereby inhibiting MPP+-induced neuronal PC12D cell death (4).
		Compound	Isoliquiritigenin	Dopaminergic cell line	In vitro	Neuroprotective effect	Protecting dopaminergic cell under oxidative stress conditions by regulating the apoptotic process (5).
		Extracts (water, in combination with *Vicia faba* and *Uncaria rhyncophylla*)	Phenolic compounds, namely, gallic acid, catechin, epicatechin, and resveratrol	Hypoe22 cell, Striatum specimens from SD rats	In-vitro, Ex vivo	Antioxidant effects and neuroprotective effects	Contrasting the upregulated lactate dehydrogenase and nitrite levels and in reducing striatal dopamine turnover (6)
Huang qi	*Astragalus mongholicus*	Compound	Astragaloside IV	Long culture-induced	In vivo and in vitro,	Preventing dopaminergic	Inhibiting of astrocyte senescence through promoting mitophagy (7).

Bunge			replicative senescence model and LPS/MPP+-induced premature senescence model and MPTP-induced PD mouse model	intraperitoneal injection	neurodegeneration in PD	
	Compound	Astragaloside IV	MPTP-induced PD mouse model, LPS-induced BV2 microglia cell	In vivo and in vitro, oral gavage	Anti-inflammatory, antioxidant, and neuroprotective effects	Protecting dopaminergic neuron from neuroinflammation and oxidative stress via activating the Nrf2 pathways and suppressing nfκb/NLRP3 inflammasome signalling pathway (8).
	Compound	Astragaloside IV	6-OHDA-treated SH-SY5Y cell	In vitro	Anti-inflammatory, antioxidant, and neuroprotective effects	Enhancing the cell viability, and inhibiting apoptosis, inflammation and oxidative stress of 6-OHDA-treated SH-SY5Y cell via activating the JAK2/STAT3 signalling pathway (9).
	Compound	Astragaloside IV	6-OHDA treated nigral cell cultures	In vitro	Neuroprotective and neurosprouting effects	Protecting dopaminergic neurons against 6-OHDA-induced degeneration, promoting neurite outgrowth and increased TH and NOS immunoreactive of dopaminergic neurons (10).
	Compound	Astragaloside IV	H_2O_2-exposed SH-SY5Y cell	In vitro	Neuroprotective effect	Inhibiting the expression of the α-synuclein via the p38 MAPK signalling pathway (11).
	Compound	Astragalus polysaccharide	6-OHDA-treated PC12 cell	In vitro	Anti-Parkinson effect	Increasing autophagy via inhibiting PI3K protein to activate PI3K/AKT/mtor pathway (12)
	Compound	Calycosin	MPTP-induced PD mouse model,	Intracerebroventricularly injection	Anti-parkinsonism and anti-	Mitigating PD symptoms through TLR/NF-κb and MAPK pathways in mice and cell lines (13)

				LPS-induced BV2 microglia cell		neuroinflammatory effects	
Bai zhu	*Atractylodes macrocephala* Koidz.	Compound	Atractylenolide-I	LPS-stimulated BV-2 cell, MPTP-intoxicated C57BL6/J mouse model	In vivo and in vitro, Intraperitoneal administration	Anti-neuroinflammatory	Abating the nuclear translocation of NF-κb and by inducing HO-1 (in vitro). Reversing MPTP-induced behavioural deficits, decreasing microglial activation, and conferring protection to dopaminergic neurons (in vivo) (14).
		Compound	Atractylenolide-I	MPP^+-induced cytotoxicity in SH-SY5Y cell	In vitro	Anti-apoptosis and antioxidant effects	Reducing pro-apoptotic signals and also by induction of antioxidant protein (15).
		Compound	Atractylon	MPTP-induced PD mouse model, SY-SY5Y cell	In vivo and in vitro, oral administration	Anti-Parkinson effect	Activating DRD2, attenuating motor deficits and gait disorders, and protecting dopaminergic neurons in MPTP-induced PD mice (16).
		Extracts (ethanol)	Atractylenolide I, atractylenolide III, and atractylodin	LPS-stimulated microglial BV2 cell	In vitro	Anti-neuroinflammatory	Attenuating the production of NO and inflammatory cytokines induced by LPS, also inhibiting the expression of inos and COX-2 without causing cytotoxicity, attenuating the transcriptional activities of NF-κb and MAPK phosphorylation, and induced HO-1 expression (17).
Dang gui	*Angelica sinensis* (Oliv.) Diels	Compound	*N*-Butylidenephthalide	MPTP-induced PD mouse model, adipose-derived stem cell	In vitro	Anti-inflammatory and neuroprotective effects	Stimulating adipose-derived stem cell with n-Butylidenephthalide improved PD recovery efficiency (18).
		Compound	*N*-Butylidenephthalide	*Caenorhabditis elegans* PD model	Mixed with the OP50/NGM plates for animal incubation	Antiparkinsonian activities	Blocking egl-1 expression to inhibit apoptosis pathways and by raising rpn-6 expression to enhance the activity of proteasomes (19).
Di huang	*Rehmannia glutinosa*	Compound	Catalpol	H_2O_2-injured astrocytes	In vitro	Antioxidant and neuroprotective	Increasing the cell viability and reducing the intracellular ROS

	(Gaertn.) DC.					effects	formation; attenuating H_2O_2-induced oxidative stress via preventing the decrease in the activities of antioxidant enzymes in glutathione redox cycling such as glutathione peroxidase, glutathione reductase and glutathione content (20).
		Compound	Catalpol	LPS-induced microglia	In vitro	Antioxidant, anti-inflammatory and neuroprotective effects	Inhibiting microglial activation and reducing the production of proinflammatory factors (21).
		Compound	Catalpol	MPTP-induced astrocytes	In vitro	Neuroprotective effect	Attenuating mitochondrial dysfunction and MAO-B activity (22).
		Compound (*Dihuang* Granule)	*Quercetin*, *kaempferol*, *luteolin*, *tanshinone IIA*, *yohimbine*, *salviolone*, *beta-sitosterol*, *4-methylenemiltirone*, *dihydrotanshinlactone*, and *2-isopropyl-8-methylphenanthrene-3,4-dione*, etc.	6-OHDA-induced PD rat model	Intragastric administration	Anti-Parkinson therapeutic effect	Modulating apoptosis through MAPK signalling (23).
		Compound (*Dihuang* Granule)	N/A	MPTP-induced PD mouse model	Oral administration	Improving gut microbial in PD	Blocking the pathway of TLR4/NF-κb (24).
		Extract (water, in formula *Liuwei dihuang*)	5-hydroxymethyl-2-furaldehyde, morroniside, loganin, paeoniflorin, verbascoside and paeonol	MPTP-induced PD mouse model, MPP^+-treated primary mesencephalic neurons	In vitro, Intraperitoneal administration	Dopaminergic neurons protection effects	Protecting dopaminergic neurons through enhancing antioxidant defense and decreasing apoptotic death (25).
Bai shao	*Paeonia lactiflora* Pall.	Compounds	Total Glucosides	MPTP-induced mouse model	Intragastric administration	Neuroprotective effect	Regulating LRRK2/alpha-synuclein signalling
		Compound	Paeoniflorin	MPTP-induced	Intragastric	Neuroprotective	Regulating the α-synuclein/PKC-δ

				PD mouse model	administration	effect	signalling pathway to reduce neuronal apoptosis (26).
		Compound	Paeoniflorin	MPP^+-induced PC12 cell	In vitro	Neuroprotective effect	Regulating mitochondrial membrane potential and Bcl-2/Bax/caspase-3 signalling pathways (27).
		Compound	Paeoniflorin	MPP^+-induced dopaminergic neurons	In vitro	Neuroprotective and anti-ferroptosis effects	Preventing ferroptosis via activation of the Akt/Nrf2/Gpx4 pathway in vitro (28).
		Compound	Paeoniflorin	MPTP-induced PD mouse model	Subcutaneous administration	Anti-neuroinflammatory effect	Inhibiting neuroinflammation by activation of the adenosine A1 receptor (29).
		Compound	Paeoniflorin	6-OHDA-induced PC12 cell	In vitro	Antioxidant and anti-apoptosis effects	Inhibiting reactive oxygen species (ROS)/pkcδ/NF-κb signalling pathway (30).
		Compound	Paeoniflorin	Glutamate induced PC12 cell	In vitro	Neuroprotective effect	Mitochondrial membrane potential and Bcl-2/ Bax signal pathway (31).
		Extract (aqueous ethanol fraction)	Total glucosides of paeony, including paeoniflorin and albiflorin	MPTP-induced PD mouse model	Intragastric administration	Neuroprotective effect	Regulating the camp/PKA/CREB signalling pathway (32)
Chai hu	1. *Bupleurum chinense* DC. 2. *Bupleurum scorzonerifolium* Willd.	Extracts (ethanol, with *Moutan* cortex, *Angelica Dahurica* root)	N/A	MPP^+-treated SH-SY5Y cell, MPTP-induced PD mouse model	Oral administration, In vitro	Neuroprotective effect	Alleviating mitochondria damage in experimental models of PD (33). Regulating nuclear receptor-related 1 protein (34).
		Extracts (ethanol)	Saikosaponin (C, A B3 and B4)	LPS-stimulated microglial cell and LPS-intraperitoneal injected C57BL/6 mouse model	In vitro, oral administration	Anti-inflammatory effect	Suppressing NF-κb-mediated inflammatory pathways (35).
Zhi	*Citrus aurantium*	Extracts	Naringin, hesperidin,	Multistress-	Oral	Prokinetic effect	Reducing gastric residual rate and

qiao/*Zhi shi/Chen pi*	L.	(water, in combination with *Citrus reticulata* Blanco and *Bupleurum chinense* DC.)	neohesperidin, saikosaponin a, and saikosaponin b2	induced delayed gastric emptying mouse model	administration, in vitro		increasing serum levels of 5-HT, MTL and SP (36).
		Extracts (water, in combination with *Rhizoma Atractylodis*)	N/A	Loperamide-induced constipated rats	Oral administration	Anti slow transit constipation	Regulation the metabolism of caffeine and vitamin B6 (37).
Ren shen	*Panax ginseng* C. A. Mey.	Compound	Ginsenoside Re	Rotenone-induced SH-SY5Y cell and *Drosophila* model	In vitro, flies administered	Neuroprotective effect	Inducing Nrf2/heme oxygenase-1 expression and activation of the dual PI3K/AKT and ERK pathways (38).
		Compounds	Ginsenosides	MPTP-induced PD mouse model	Oral administration	Neuroprotective effect	Anti-apoptosis, antiinflammation, antioxidant, and maintenance of blood-brain barrier integrity (39).
		Compounds	Ginsenosides Rd and Re	Ccl4-induced primary dopaminergic cell	In vitro	Neuroprotective effect	Lowering oxidative stress and anti-inflammation (40).
		Compound	Ginsenoside Rb1	MPTP-induced PD mouse model	Administered intraperitoneally	Neuroprotective effect	Regulating prefrontal cortical gabaergic transmission (41).
		Compound	Ginsenoside Rg1	MPTP-induced PD mouse model	In vivo and in vitro, Intraperitoneal administration	Neuroprotective effect	Regulating the Wnt/β-catenin signalling pathway (42).
		Compound	Ginsenoside Rg1	MPTP-induced PD mouse model	Intraperitoneal administration	Immunomodulatory and neuroprotective effects	Regulating the peripheral and central inflammation (43).
		Compound	Ginsenoside Rg1	MPTP-induced PD mouse model	Intraperitoneal administration	Anti-Parkinson effect	Restoring motor functions to physiological level in MPTP-treated PD mice, and attenuating the

					MPTP-induced loss of dopaminergic neurons in the substantia nigra and striatum (44).
Compound	Ginsenoside Rg1	MPTP-induced PD mouse model	Oral administration	Neuroprotective and anti-inflammatory effects	Reducing aberrant α-synuclein-mediated neuroinflammation (45).
Compound	Ginsenoside Rg1	MPTP-induced PD mouse model	Intraperitoneal injection	Neuroprotective effect	Remitting the iron-regulated protein dyshomeostasis by ferritin and against lipid peroxidation stress in oligodendrocytes (46).
Compound	Ginsenoside Rg3	Rotenone-induced PD mouse model	Intragastric administration	Neuroprotective and antioxidant effects	Regulating glutathione cysteine ligase modulatory subunit and glutathione cysteine ligase regulatory subunit expression (47).
Extract	Ginsenosides	BSSG-induced PD mouse model	Oral administration	Neuroprotective, anti-inflammatory, anti-apoptosis and antioxidant effects	Protecting against dopaminergic neurons in the substantia nigra. Reducing indices of inflammation, apoptosis and cell stress, preventing the accumulation of insoluble α-synuclein aggregates (48).
Extract (water)	Ginsenosides Rg1, Re and Rb1	SH-SY5Y human neuroblastoma cell	In vitro	Neuroprotective and anti-cytotoxicity effects	Suppressing ROS generation and inhibiting mitochondria-dependent apoptotic pathway (49).
Extract (water)	Ginsenosides	MPTP-induced PD mouse model	Oral administration	Neuroprotective effect	Inhibiting MPTP-induced dopaminergic neuronal death and suppressing the cleavage of p35 to p25 in the substantia nigra and striatum (50).
Extract (ethanol)	Gintonin	MPTP-induced PD mouse model	Oral administration	Anti-apoptosis, anti-inflammatory and antioxidant agent effects	Activation of the nuclear factor erythroid 2-related factor 2 pathways and the inhibition of phosphorylation of the mitogen-activated protein kinases and nuclear factor-kappa B signalling pathways (51).
Extracts	Ginseng total protein	Dm $PINK_1$	Oral	Neuroprotective	Protecting against mitochondrial

				model of PD	administration	effect	dysfunction and neurodegeneration by inducing uprmt in the Dm PINK1B9 model of PD (52).
		Extract (water)	N/A	MPTP-induced PD mouse model	Oral administration	Anti-Parkinson effect	Preventing MPTP-induced leaky gut barrier, inflammation, and accumulation of asyn (53).
		Extract (water)	N/A	MPTP-induced PD mouse model	Oral administration	Anti-Parkinson effect	Regulating neuronal formation and energy metabolism for survival (54).
		Extract (water)	N/A	MPP+-induced SH-SY5Y cell	In vitro	Anti- apoptosis and anti-mitophagy effect	Regulating cytochrome c release from mitochondria and PINK1/parkin-mediated mitophagy, through regulation of the Bcl-2 family (55).
		Extract (water)	N/A	MPTP-induced mouse model	Oral administration	Anti-Parkinson effect	Suppressing dopaminergic neuronal death, augmenting the number of brdu- and brdu/double cortin (Dcx) and enhancing the expression of proliferation cell nuclear antigen, BDNF, GDNF, CDNF, CNTF, DRD3 and D5 mrnas (56).
		Extract	Pseudoginsenoside-f11	6-OHDA-induced PD rat model	Oral administration	Anti-Parkinson effect	Inhibiting free radical formation and stimulating endogenous antioxidant release (57).
Fu ling	*Poria cocos* (Schw.) Wolf	Compound	Acidic polysaccharides	CUMS rat model	Intragastric administration	Antidepressant effect	Regulating neurotransmitters and NLRP3 inflammasome signalling pathway (58).
		Compound	Pachymic Acid	Pentobarbital-induced sleep mouse model	Intragastric administration	Sedative-hypnotic effect	Enhancing pentobarbital-induced sleeping behaviours via GABAA-ergic mechanisms in rodents (59).
		Compound	Pachymic acid	Cerebral ischemia/reperfusion injury rat model	Intragastric administration	Neuroprotective effect	Activating PI3K / Akt signalling pathway(60).
		Extract (ethanol)	N/A	ACTH-induced sleep disturbed mouse model	Intragastric administration	Sedative-hypnotic effect	Improving sleep quality under a normal sleep state through the $GABA_A$ receptor; promoting and improving sleep quality and sleep

							structure in both the arousal activation state and stress- based sleep disturbance (61).
		Extract (water)	Polysaccharides	CUMS rat model	Intragastric administration	Antidepressant-like effect	Regulating monoaminergic neurotransmission (DA, 5-HT) and inactivation of inflammation (p38, NF-κb and TNF-α) (62).
Dang shen	1. *Codonopsis pilosula* Nannf.<br>2. *Codonopsis pilosula var.* Pilosula<br>3. *Campanumoea pilosula* Franch.	Compound	Isorhapontigenin	PC12 cell	In vitro	Antioxidant effect	Enhanced the antioxidant effect induced by 1-Methyl-4-phenylpyridine in PC12 cell by suppressing the activation of the PI3K/Akt signaling pathway (63).
Bu zhong yi qi tang		Extracts (water)	ferulicacid, isoferulicacid, hesperidin, calycosin, glycyrrhizicacid, aposaponind, etc.	Cerebral ischemia mouse model	Intragastric administration	Neuroprotective effect	Regulating intestinal microbiota and increasing the abundance of butyrate-producing Prevotellaceae_NK3B31_group and probiotic *Akkermansia* in mice 72h after surgery (64).
		Extracts (water)	Liquiritin apioside, Liquiritin, Nodakenin, Hesperidin, Glycyrrhizin, Decursin, Decursinol angelate	Aβ-injected mouse model	In vitro, intragastric administration	anti-dementia and neuroprotective effects	Enhancing inhibition of Aβ aggregation and BACE activity in vivo, as well as antioxidant activity in vitro, suppressing Aβ aggregation and expression, as well as expression of Aβ, NeuN, and BDNF in the hippocampi of Aβ-injected mice (65).

Note: Aβ: amyloid-β; ACTH: adrenocorticotropic hormone; aSyn: alpha-synuclein; BDNF: brain derived neurotrophic factor; BSSG: β-sitosterol β-d-glucoside; CCl4: Carbon tetrachloride; CDNF: cerebral dopamine neurotrophic factor; CNTF: ciliary neurotrophic factor; COX-2: cyclooxygenase; CUMS: chronic unpredictable mild stress; Dm: Drosophila melanogaster; GDNF: glial cell derived neurotrophic factor; DRD3: dopamine receptor D3; N/A: not available. H_2O_2: hydrogen peroxide; HO-1: heme oxygenase-1; iNOS: inducible nitric oxide synthase; SD: Sprague-Dawley; LPS/MPP+: lipopolysaccharide/1-methyl-4-phenylpyridinium; MAPK: mitogen-activated protein kinase; MPTP: 1-Methyl-4-phenyl-1,2,3,6-tetrahydropyridine; NF-κB: nuclear factor-κB; NO: nitric oxide; NOS: nitrite oxide synthase; PC12: pheochromocytoma cell; PD: Parkinson's disease; $PINK_1$: PTEN-induced putative kinase 1; ROS: reactive oxygen specie; SH-SY5Y cell: SK-N-SH neuroblastoma cell line; TH: tyrosine hydrolase;

1. Karthikkeyan G, Prabhu A, Pervaje R, Pervaje SK, Modi PK, Prasad TSK. Data on dose-dependent cytotoxicity of rotenone and neuroprotection conferred by Yashtimadhu (Glycyrrhiza glabra L.) in an in vitro Parkinson's disease model. Data Brief. 2021;39:107535.

2. Karthikkeyan G, Behera SK, Upadhyay SS, Pervaje R, Prasad TSK, Modi PK. Metabolomics analysis highlights Yashtimadhu (Glycyrrhiza glabra L.)-mediated neuroprotection in a rotenone-induced cellular model of Parkinson's disease by restoring the mTORC1-AMPK1 axis in autophagic regulation. Phytother Res. 2022;36(5):2207-22.

3. Ramadan S, Sabry MM, Saad MA, Angeloni S, Sabry OM, Caprioli G, et al. Dismantling Parkinson's disease with herbs: MAO-B inhibitory activity and quantification of chemical constituents using HPLC-MS/MS of Egyptian local market plants. Nat Prod Res. 2022;36(22):5766-71.

4. Fujimaki T, Saiki S, Tashiro E, Yamada D, Kitagawa M, Hattori N, et al. Identification of licopyranocoumarin and glycyrurol from herbal medicines as neuroprotective compounds for Parkinson's disease. PLoS One. 2014;9(6):e100395.

5. Hwang CK, Chun HS. Isoliquiritigenin isolated from licorice Glycyrrhiza uralensis prevents 6-hydroxydopamine-induced apoptosis in dopaminergic neurons. Biosci Biotechnol Biochem. 2012;76(3):536-43.

6. Orlando G, Chiavaroli A, Leone S, Brunetti L, Politi M, Menghini L, et al. Inhibitory Effects Induced by Vicia faba, Uncaria rhyncophylla, and Glycyrrhiza glabra Water Extracts on Oxidative Stress Biomarkers and Dopamine Turnover in HypoE22 Cell and Isolated Rat Striatum Challenged with 6-Hydroxydopamine. Antioxidants (Basel). 2019;8(12).

7. Xia ML, Xie XH, Ding JH, Du RH, Hu G. Astragaloside IV inhibits astrocyte senescence: implication in Parkinson's disease. J Neuroinflammation. 2020;17(1):105.

8. Yang C, Mo Y, Xu E, Wen H, Wei R, Li S, et al. Astragaloside IV ameliorates motor deficits and dopaminergic neuron degeneration via inhibiting neuroinflammation and oxidative stress in a Parkinson's disease mouse model. Int Immunopharmacol. 2019;75:105651.

9. Xu Z, Yang D, Huang X, Huang H. Astragaloside IV Protects 6-Hydroxydopamine-Induced SH-SY5Y Cell Model of Parkinson's Disease via Activating the JAK2/STAT3 Pathway. Front Neurosci. 2021;15:631501.

10. Chan WS, Durairajan SS, Lu JH, Wang Y, Xie LX, Kum WF, et al. Neuroprotective effects of Astragaloside IV in 6-hydroxydopamine-treated primary nigral cell culture. Neurochem Int. 2009;55(6):414-22.

11. Liu X, Zhang J, Wang S, Qiu J, Yu C. Astragaloside IV attenuates the H2O2-induced apoptosis of neuronal cell by inhibiting α-synuclein expression via the p38 MAPK pathway. Int J Mol Med. 2017;40(6):1772-80.

12. Tan Y, Yin L, Sun Z, Shao S, Chen W, Man X, et al. Astragalus polysaccharide exerts anti-Parkinson via activating the PI3K/AKT/mTOR pathway to increase cellular autophagy level in vitro. Int J Biol Macromol. 2020;153:349-56.

13. Yang J, Jia M, Zhang X, Wang P. Calycosin attenuates MPTP-induced Parkinson's disease by suppressing the activation of TLR/NF-κB and MAPK pathways. Phytother Res. 2019;33(2):309-18.

14. More S, Choi DK. Neuroprotective Role of Atractylenolide-I in an In Vitro and In Vivo Model of Parkinson's Disease. Nutrients. 2017;9(5).

15. More SV, Choi DK. Atractylenolide-I Protects Human SH-SY5Y Cell from 1-Methyl-4-Phenylpyridinium-Induced Apoptotic Cell Death. Int J Mol Sci. 2017;18(5).

16. Li H, Wang F, Zhou Z, Jiang X, Li F, Feng Y, et al. Atractylon, a novel dopamine 2 receptor agonist, ameliorates Parkinsonian like motor dysfunctions in MPTP-induced mice. NeuroToxicology. 2022;89:121-6.

17. Jeong YH, Li W, Go Y, Oh YC. Atractylodis Rhizoma Alba Attenuates Neuroinflammation in BV2 Microglia upon LPS Stimulation by Inducing HO-1 Activity and Inhibiting NF-κB and MAPK. Int J Mol Sci. 2019;20(16).

18. Chi K, Fu RH, Huang YC, Chen SY, Hsu CJ, Lin SZ, et al. Adipose-derived Stem Cell Stimulated with n-Butylidenephthalide Exhibit Therapeutic Effects in a Mouse Model of Parkinson's Disease. Cell Transplant. 2018;27(3):456-70.

19. Fu RH, Harn HJ, Liu SP, Chen CS, Chang WL, Chen YM, et al. n-butylidenephthalide protects against dopaminergic neuron degeneration and α-synuclein accumulation in Caenorhabditis elegans models of Parkinson's disease. PLoS One. 2014;9(1):e85305.

20. Bi J, Jiang B, Liu JH, Lei C, Zhang XL, An L-J. Protective effects of catalpol against H2O2-induced oxidative stress in astrocytes primary cultures. Neuroscience Letters. 2008;442(3):224-7.

21. Tian YY, An LJ, Jiang L, Duan YL, Chen J, Jiang B. Catalpol protects dopaminergic neurons from LPS-induced neurotoxicity in mesencephalic neuron-glia cultures. Life Sci. 2006;80(3):193-9.

22. Bi J, Wang XB, Chen L, Hao S, An LJ, Jiang B, et al. Catalpol protects mesencephalic neurons against MPTP induced neurotoxicity via attenuation of mitochondrial dysfunction and MAO-B activity. Toxicol In Vitro. 2008;22(8):1883-9.

23. Wang L, Yang YF, Chen L, He ZQ, Bi DY, Zhang L, et al. Compound Dihuang Granule Inhibits Nigrostriatal Pathway Apoptosis in Parkinson's Disease by Suppressing the JNK/AP-1 Pathway. Front Pharmacol. 2021;12:621359.

24. He ZQ, Huan PF, Wang L, He JC. Compound Dihuang Granule Changes Gut Microbiota of MPTP-Induced Parkinson's Disease Mice via Inhibiting TLR4/NF-κB Signaling. Neurochem Res. 2023;48(12):3610-24.

25. Tseng YT, Chang FR, Lo YC. The Chinese herbal formula Liuwei dihuang protects dopaminergic neurons against Parkinson's toxin through enhancing antioxidative defense and preventing apoptotic death. Phytomedicine. 2014;21(5):724-33.

26. Guo K, Zhang Y, Li L, Zhang J, Rong H, Liu D, et al. Neuroprotective effect of paeoniflorin in the mouse model of Parkinson's disease through α-synuclein/protein kinase C δ subtype signaling pathway. Neuroreport. 2021;32(17):1379-87.

27. Zheng M, Liu C, Fan Y, Shi D, Zhang Y. Protective Effects of Paeoniflorin Against MPP(+)-induced Neurotoxicity in PC12 Cell. Neurochem Res. 2016;41(6):1323-34.

28. Wang L, An H, Yu F, Yang J, Ding H, Bao Y, et al. The neuroprotective effects of paeoniflorin against MPP(+)-induced damage to dopaminergic neurons via the Akt/Nrf2/GPX4 pathway. J Chem Neuroanat. 2022;122:102103.

29. Liu HQ, Zhang WY, Luo XT, Ye Y, Zhu XZ. Paeoniflorin attenuates neuroinflammation and dopaminergic neurodegeneration in the MPTP model of Parkinson's disease by activation of adenosine A1 receptor. Br J Pharmacol. 2006;148(3):314-25.

30. Dong H, Li R, Yu C, Xu T, Zhang X, Dong M. Paeoniflorin inhibition of 6-hydroxydopamine-induced apoptosis in PC12 cell via suppressing reactive oxygen species-mediated PKCδ/NF-κB pathway. Neuroscience. 2015;285:70-80.

31. Sun R, Wang K, Wu D, Li X, Ou Y. Protective effect of paeoniflorin against glutamate-induced neurotoxicity in PC12 cell via Bcl-2/Bax signal pathway. Folia Neuropathol. 2012;50(3):270-6.

32. Zheng M, Liu C, Fan Y, Shi D, Jian W. Total glucosides of paeony (TGP) extracted from Radix Paeoniae Alba exerts neuroprotective effects in MPTP-induced experimental parkinsonism by regulating the cAMP/PKA/CREB signaling pathway. J Ethnopharmacol. 2019;245:112182.

33. Jeong JS, Piao Y, Kang S, Son M, Kang YC, Du XF, et al. Triple herbal extract DA-9805 exerts a neuroprotective effect via amelioration of mitochondrial damage in experimental models of Parkinson's disease. Sci Rep. 2018;8(1):15953.

34. Sim Y, Park G, Eo H, Huh E, Gu PS, Hong SP, et al. Protective effects of a herbal extract combination of Bupleurum falcatum, Paeonia suffruticosa, and Angelica dahurica against MPTP-induced neurotoxicity via regulation of nuclear receptor-related 1 protein. Neuroscience. 2017;340:166-75.

35. Park WH, Kang S, Piao Y, Pak CJ, Oh MS, Kim J, et al. Ethanol extract of Bupleurum falcatum and saikosaponins inhibit neuroinflammation via inhibition of NF-κB. Journal of ethnopharmacology. 2015;174:37-44.

36. Gong Y, Liang X, Dai Y, Huang X, Su Q, Ma Y, et al. Prokinetic effects of Citrus reticulata and Citrus aurantium extract with/without Bupleurum chinense using multistress-induced delayed gastric emptying models. Pharm Biol. 2023;61(1):345-55.

37. Yan S, Hao M, Yang H, Sun M, Wu B, Yue Y, et al. Metabolomics study on the therapeutic effect of the Chinese herb pair Fructus Aurantii Immaturus and Rhizoma Atractylodis Macrocephalae in constipated rats based on UPLC-Q/TOF-MS analysis. Ann Palliat Med. 2020;9(5):2837-52.

38. Qiao J, Zhao Y, Liu Y, Zhang S, Zhao W, Liu S, et al. Neuroprotective effect of Ginsenoside Re against neurotoxin-induced Parkinson's disease models via induction of Nrf2. Mol Med Rep. 2022;25(6).

39. Choi JH, Jang M, Nah SY, Oh S, Cho IH. Multitarget effects of Korean Red Ginseng in animal model of Parkinson's disease: antiapoptosis, antioxidant, antiinflammation, and maintenance of blood-brain barrier integrity. J Ginseng Res. 2018;42(3):379-88.

40. Zhang X, Wang Y, Ma C, Yan Y, Yang Y, Wang X, et al. Ginsenoside Rd and ginsenoside Re offer neuroprotection in a novel model of Parkinson's disease. Am J Neurodegener Dis. 2016;5(1):52-61.

41. Liu Y, Zong X, Huang J, Guan Y, Li Y, Du T, et al. Ginsenoside Rb1 regulates prefrontal cortical GABAergic transmission in MPTP-treated mice. Aging (Albany NY). 2019;11(14):5008-34.

42. Zhou T, Zu G, Zhang X, Wang X, Li S, Gong X, et al. Neuroprotective effects of ginsenoside Rg1 through the Wnt/β-catenin signaling pathway in both in vivo and in vitro models of Parkinson's disease. Neuropharmacology. 2016;101:480-9.

43. Zhou TT, Zu G, Wang X, Zhang XG, Li S, Liang ZH, et al. Immunomodulatory and neuroprotective effects of ginsenoside Rg1 in the MPTP(1-methyl-4-phenyl-1,2,3,6-tetrahydropyridine) -induced mouse model of Parkinson's disease. Int Immunopharmacol. 2015;29(2):334-43.

44. Jiang W, Wang Z, Jiang Y, Lu M, Li X. Ginsenoside Rg1 Ameliorates Motor Function in an Animal Model of Parkinson's Disease. Pharmacology. 2015;96(1-2):25-31.

45. Heng Y, Zhang QS, Mu Z, Hu JF, Yuan YH, Chen NH. Ginsenoside Rg1 attenuates motor impairment and neuroinflammation in the MPTP-probenecid-induced parkinsonism mouse model by targeting α-synuclein abnormalities in the substantia nigra. Toxicol Lett. 2016;243:7-21.

46. Chen Y, Li YY, Wang S, Zhou T, Chen NH, Yuan YH. Ginsenoside Rg1 Plays a Neuroprotective Role in Regulating the Iron-Regulated Proteins and Against Lipid Peroxidation in Oligodendrocytes. Neurochem Res. 2022;47(6):1721-35.

47. Han Y, Wang T, Li C, Wang Z, Zhao Y, He J, et al. Ginsenoside Rg3 exerts a neuroprotective effect in rotenone-induced Parkinson's disease mice via its anti-oxidative properties. European Journal of Pharmacology. 2021;909:174413.

48. Van Kampen JM, Baranowski DB, Shaw CA, Kay DG. Panax ginseng is neuroprotective in a novel progressive model of Parkinson's disease. Experimental Gerontology. 2014;50:95-105.

49. Hu S, Han R, Mak S, Han Y. Protection against 1-methyl-4-phenylpyridinium ion (MPP+)-induced apoptosis by water extract of ginseng (Panax ginseng C.A. Meyer) in SH-SY5Y cell. J Ethnopharmacol. 2011;135(1):34-42.

50. Jun YL, Bae CH, Kim D, Koo S, Kim S. Korean Red Ginseng protects dopaminergic neurons by suppressing the cleavage of p35 to p25 in a Parkinson's disease mouse model. J Ginseng Res. 2015;39(2):148-54.

51. Choi JH, Jang M, Oh S, Nah SY, Cho IH. Multi-Target Protective Effects of Gintonin in 1-Methyl-4-phenyl-1,2,3,6-tetrahydropyridine-Mediated Model of Parkinson's Disease via Lysophosphatidic Acid Receptors. Front Pharmacol. 2018;9:515.

52. Liu M, Yu S, Wang J, Qiao J, Liu Y, Wang S, et al. Ginseng protein protects against mitochondrial dysfunction and neurodegeneration by inducing mitochondrial unfolded protein response in Drosophila melanogaster PINK1 model of Parkinson's disease. J Ethnopharmacol. 2020;247:112213.

53. Jeon H, Kim HY, Bae CH, Lee Y, Kim S. Korean Red Ginseng Regulates Intestinal Tight Junction and Inflammation in the Colon of a Parkinson's Disease Mouse Model. J Med Food. 2020;23(12):1231-7.

54. Kim D, Kwon S, Jeon H, Ryu S, Ha KT, Kim S. Proteomic change by Korean Red Ginseng in the substantia nigra of a Parkinson's disease mouse model. J Ginseng Res. 2018;42(4):429-35.

55. Jeon H, Kim HY, Bae CH, Lee Y, Koo S, Kim S. Korean red ginseng decreases 1-methyl-4-phenylpyridinium-induced mitophagy in SH-SY5Y cell. J Integr Med. 2021;19(6):537-44.

56. Ryu S, Jeon H, Koo S, Kim S. Korean Red Ginseng Enhances Neurogenesis in the Subventricular Zone of 1-Methyl-4-Phenyl-1,2,3,6-Tetrahydropyridine-Treated Mice. Front Aging Neurosci. 2018;10:355.

57. Wang JY, Yang JY, Wang F, Fu SY, Hou Y, Jiang B, et al. Neuroprotective effect of pseudoginsenoside-f11 on a rat model of Parkinson's disease induced by 6-hydroxydopamine. Evid Based Complement Alternat Med. 2013;2013:152798.

58. Chen KZ, Chen S, Ren JY, Lin S, Xiao MJ, Cheng L, et al. [Antidepressant effect of acidic polysaccharides from Poria and their regulation of neurotransmitters and NLRP3 pathway]. Zhongguo Zhong Yao Za Zhi. 2021;46(19):5088-95.

59. Shah VK, Choi JJ, Han JY, Lee MK, Hong JT, Oh KW. Pachymic Acid Enhances Pentobarbital-Induced Sleeping Behaviors via GABAA-ergic Systems in Mice. Biomol Ther (Seoul). 2014;22(4):314-20.

60. Pang Y, Zhu S, Pei H. Pachymic acid protects against cerebral ischemia/reperfusion injury by the PI3K/Akt signaling pathway. Metab Brain Dis. 2020;35(4):673-80.

61. Kim H, Park I, Park K, Park S, Kim YI, Park BG. The Positive Effects of Poria cocos Extract on Quality of Sleep in Insomnia Rat Models. Int J Environ Res Public Health. 2022;19(11).

62. Huang YJ, Hsu NY, Lu KH, Lin YE, Lin SH, Lu YS, et al. Poria cocos water extract ameliorates the behavioral deficits induced by unpredictable chronic mild stress in rats by down-regulating inflammation. J Ethnopharmacol. 2020;258:112566.

63. Gu Y, Wang T, Chen J, Zhou Z, Wang Y, Chen J, et al. The Chinese Herb Codonopsis pilosula Isolate Isorhapontigenin protects against oxidative stress injury by inhibiting the activation of PI3K/Akt signaling pathway. J Integr Neurosci. 2020;19(2):333-40.

64. Li Q, Cao M, Wei Z, Mei J, Zhang Y, Li M, et al. The protective effect of Buzhong Yiqi decoction on ischemic stroke mice and the mechanism of gut microbiota. Front Neurosci. 2022;16:956620.

65. Lim HS, Kim YJ, Sohn E, Yoon J, Kim BY, Jeong SJ. Bojungikgi-Tang, a Traditional Herbal Formula, Exerts Neuroprotective Effects and Ameliorates Memory Impairments in Alzheimer's Disease-Like Experimental Models. Nutrients. 2018;10(12).
